# Fluorescence-tagged salivary small extracellular vesicles as a nanotool in early diagnosis of Parkinson’s disease

**DOI:** 10.1186/s12916-023-03031-1

**Published:** 2023-09-04

**Authors:** Simran Rastogi, Komal Rani, Sanskriti Rai, Rishabh Singh, Prahalad Singh Bharti, Vaibhav Sharma, Jyoti Sahu, Vrinda Kapoor, Poorvi Vishwakarma, Sumit Garg, Shivajirao Lahu Gholap, Krishna Kishore Inampudi, Gyan Prakash Modi, Neerja Rani, Madhavi Tripathi, Achal Srivastava, Roopa Rajan, Fredrik Nikolajeff, Saroj Kumar

**Affiliations:** 1 Department of Biophysics, All India Institute of Medical Sciences, New Delhi, 110029 India; 2 Department of Pathology & Laboratory Medicine, All India Institute of Medical Sciences Bibinagar, Hyderabad, 508126 India; 3 Department of Health, Education, and Technology, Luleå University of Technology, 97187 Luleå, Sweden; 4 School of Interdisciplinary Research, Indian Institute of Technology Delhi, New Delhi, 110016 India; 5 Department of Nuclear Medicine, All India Institute of Medical Sciences, New Delhi, 110029 India; 6 Department of Chemistry, Indian Institute of Technology Delhi, New Delhi, 110016 India; 7 Department of Pharmaceutical Engineering & Technology, Indian Institute of Technology BHU, Varanasi, 221005 India; 8 Department of Anatomy, All India Institute of Medical Sciences, New Delhi, 110029 India; 9 Department of Neurology, All India Institute of Medical Sciences, New Delhi, 110029 India

**Keywords:** Parkinson’s disease, Saliva, Small extracellular vesicles, Alpha-synuclein, Nanoparticle tracking analysis, Prodromal, TRODAT scan

## Abstract

**Background:**

Parkinson’s disease is generally asymptomatic at earlier stages. At an early stage, there is an extensive progression in the neuropathological hallmarks, although, at this stage, diagnosis is not possible with currently available diagnostic methods. Therefore, the pressing need is for susceptibility risk biomarkers that can aid in better diagnosis and therapeutics as well can objectively serve to measure the endpoint of disease progression. The role of small extracellular vesicles (sEV) in the progression of neurodegenerative diseases could be potent in playing a revolutionary role in biomarker discovery.

**Methods:**

In our study, the salivary sEV were efficiently isolated by chemical precipitation combined with ultrafiltration from subjects (PD = 70, healthy controls = 26, and prodromal PD = 08), followed by antibody-based validation with CD63, CD9, GAPDH, Flotillin-1, and L1CAM. Morphological characterization of the isolated sEV through transmission electron microscopy. The quantification of sEV was achieved by fluorescence (lipid-binding dye-labeled) nanoparticle tracking analysis and antibody-based (CD63 Alexa fluor 488 tagged sEV) nanoparticle tracking analysis. The total alpha-synuclein (α-syn_Total_) in salivary sEVs cargo was quantified by ELISA. The disease severity staging confirmation for *n* = 18 clinically diagnosed Parkinson’s disease patients was done by ^99m^Tc-TRODAT-single-photon emission computed tomography.

**Results:**

We observed a significant increase in total sEVs concentration in PD patients than in the healthy control (HC), where fluorescence lipid-binding dye-tagged sEV were observed to be higher in PD (*p* = 0.0001) than in the HC using NTA with a sensitivity of 94.34%. In the prodromal PD cases, the fluorescence lipid-binding dye-tagged sEV concentration was found to be higher (*p* = 0.008) than in HC. This result was validated through anti-CD63 tagged sEV (*p* = 0.0006) with similar sensitivity of 94.12%. We further validated our findings with the ELISA based on α-syn_Total_ concentration in sEV, where it was observed to be higher in PD (*p* = 0.004) with a sensitivity of 88.24%. The caudate binding ratios in ^99m^Tc-TRODAT-SPECT represent a positive correlation with sEV concentration (*r* = 0.8117 with *p* = 0.0112).

**Conclusions:**

In this study, for the first time, we have found that the fluorescence-tagged sEV has the potential to screen the progression of disease with clinically acceptable sensitivity and can be a potent early detection method for PD.

**Graphical Abstract:**

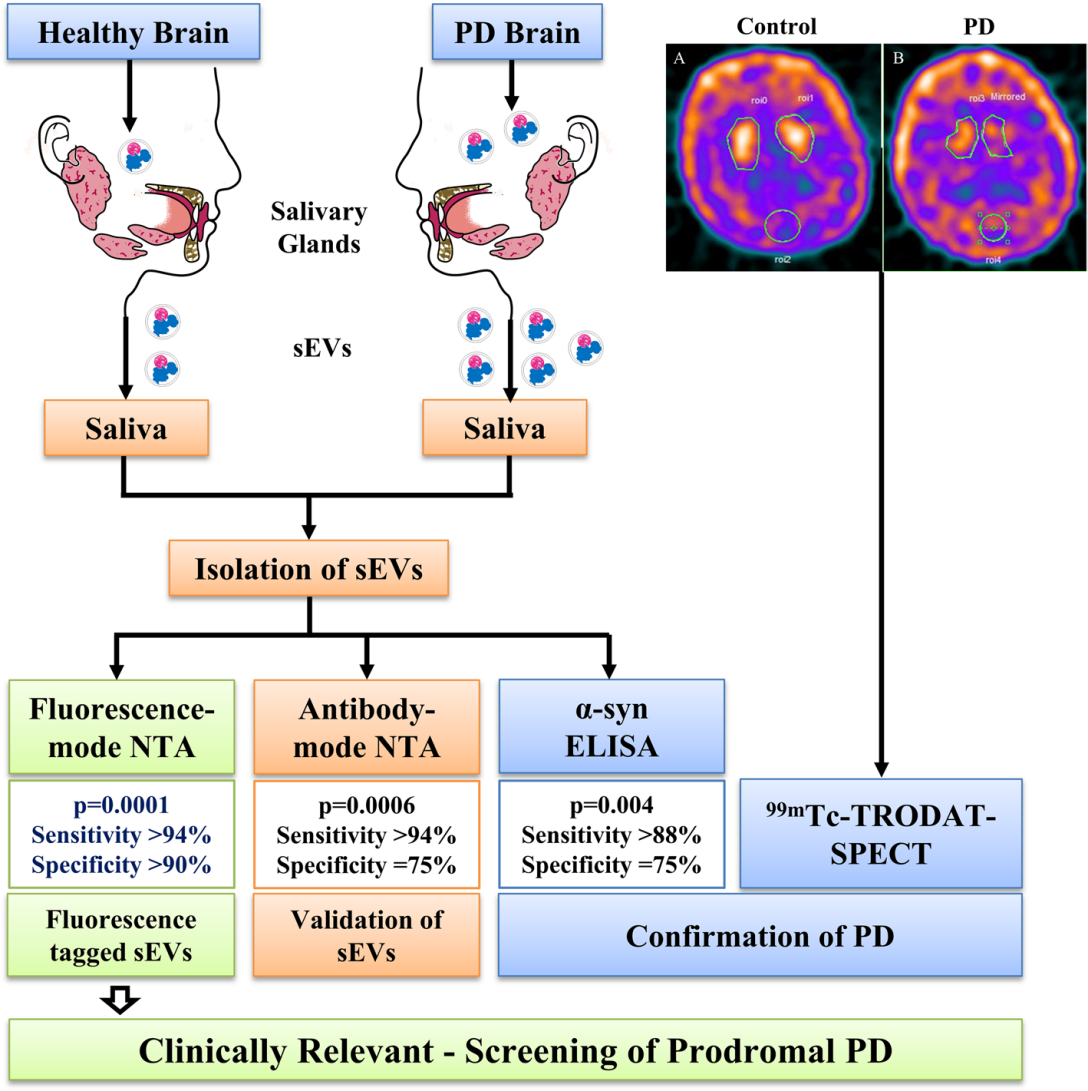

**Supplementary Information:**

The online version contains supplementary material available at 10.1186/s12916-023-03031-1.

## Background

Parkinson’s disease (PD) accounts for the second-most prevalent neurodegenerative disorder [1]. The neuropathophysiology of PD portrays major characteristics; the loss of dopaminergic neurons in the ventrolateral portions of substantia nigra pars compacta of the midbrain [2, 3], the intraneuronal aggregates of misfolded alpha-synuclein (α-syn) proteins as well as the presence of Lewy bodies [4, 5]. The crucial connecting link between the α-syn and disrupted dopamine metabolism is still elusive. Individuals affected with PD show a series of motor and non-motor symptoms. PD is considered a manageable neurodegenerative disorder. However, as of now, the disease progression cannot be stopped, and the disability worsens with time. The need of the hour is to develop an effective diagnostic methodology in the early stages that would eventually lead to improved prognosis and better therapeutics.

Recently, the clinical utility of susceptibility-risk biomarkers that possess the potential to identify a developing disease condition in its early stages has largely taken a surge. Some CSF and blood-based molecular markers such as α-syn, DJ-1, tau protein, and miRNAs are studied. Several studies have also identified the total α-syn concentration in CSF; however, several limitations hinder its usage in the development of an early diagnosis method. Among many factors, the heterogeneity in the sample collection, invasiveness of the collection protocol, and the contamination by blood, which sometimes leads to higher levels of α-syn are some known limitations [6]. Furthermore, some studies showed that the levels of total alpha-synuclein concentration were increased in the peripheral blood of Parkinson’s disease in comparison to the healthy control. In a study by Shi et al. (2014), they suggested an increased efflux of α-synuclein to the peripheral blood in PD patients, as well as, a correlation between the plasma sEV α-synuclein expression and disease severity was reported (*r* = 0.176, *p* = 0.004, Pearson correlation) [7]. In another study by Jiang et al. (2021), α-synuclein level in L1CAM-immunocaptured sEVs from serum samples was reported to be more significant in PD patients than controls, as well as, can be utilized in differentiation between PD and other tauopathies [8]. Similarly, Dutta et al. (2021) reported α-syn in blood sEVs can differentiate PD with MSA [9]. In the case of blood and saliva-based markers, many studies have been reported, out of which some indicate high levels of α-syn_total_ and α-syn_oligo_; however, some of them indicate no change between control and PD patients [6, 10–13]. sEV is now known to facilitate intercellular communication and propagation of disease pathologies. In PD, where the dysfunction of the lysosomal autophagy system (LAS) interferes with the degradation of α-syn, the studies have shown an increased secretion of sEV in the extracellular spaces [14–16]. The emerging role of sEV cargo and the variability of its content as per the targeted and recipient cells have made it a prospective biomarker candidate [10, 17]. Over the years, much emphasis has been laid on developing methodologies with high diagnostic accuracy. In this regard, some studies were attempted based on α-syn_total_ (alpha-synuclein total) and α-syn_oligo_ (alpha-synuclein oligomer) in the plasma and salivary sEV cargo [18–21]. These studies either failed to achieve clinical acceptability. One study has shown the potential of α-syn_oligo_ in plasma samples of PD, however, they only focused on neural-derived EVs as well as had a very small sample size [22]. Saliva contains various biological information like blood and can be utilized as a potential diagnostic tool due to its advantages over blood/CSF-like noninvasive method of sample collection with ease, stability, and similar content profile as other biofluids [23, 24]. However, there are only a few studies available showing the role of salivary sEVs in PD [12, 13], and research is still in a nascent stage. Therefore, the objective of the present work is to propose an easy and cost-effective quantification method based on saliva-derived sEV with high sensitivity and specificity that can be clinically acceptable.

## Methods

### Subjects

The total sample size of the study was 104. A total of *n* = 70 Parkinson’s disease patients were recruited from the Department of Neurology, All India Institute of Medical Sciences, New Delhi, India. The PD subjects used in the study are clinically diagnosed PD cases, which are cases of clinical definite PD. The clinical diagnosis was performed by the movement disorder specialist in the neurology department. UPDRS (Unified Parkinson’s Disease Rating Scale) and MMSE (Mini-Mental State Examination) were used for preliminary evaluation. A total of *n* = 26 age-matched controls and *n* = 8 prodromal PD were recruited for the study. ^99m^Tc-TRODAT-SPECT/CT was performed at the Department of Nuclear Medicine for *n* = 18 Parkinson’s patients and *n* = 23 healthy age-matched controls.

### Study ethical approval

The ethical clearance was obtained from the institutional ethics committee, All Institute of Medical Sciences, New Delhi, India. The ethical clearance number for this study is IEC- 287/07.05.2021, RP-05/2021. All the subjects were recruited for the study after obtainment of the written informed consent form. A detailed written participant information sheet and participant informed consent form were provided to the subjects to take part in this study and their signatures were obtained.

### Sample collection

For the collection of saliva samples, all the necessary precautions were followed. Two milliliters of unstimulated saliva sample was collected in the resting position from the floor of the mouth. Throughout the collection process, the vials containing the samples were kept on ice. The obtained saliva samples were further subjected to centrifugation at 1700 g for 20 min at 4 °C to remove the cell debris as a pellet and collected the supernatant. Then it was followed by centrifugation at 10,000 g for 20 min at 4 °C to get the clear saliva samples as supernatant. Finally, the clarified saliva samples were kept at 4 °C for further experiments and − 80 °C for long-term storage. The clarified saliva samples stored at − 80 °C before usage were freeze-thawed on ice for 2 h, followed by a final clarification centrifugation step at 10,000 g to obtain a clear supernatant for further processing. It is important to emphasize that at no point during the collection and processing procedures did the saliva samples experience temperatures above 4 °C.

### Isolation of pure sEV by chemical-based precipitation combined with ultrafiltration

The sEVs were isolated from the saliva samples of PD patients and age-matched healthy controls through chemical-based precipitation combined with ultrafiltration. An equal volume (180 µl) of clarified saliva samples (through serial centrifugations) was used from each patient as well as control samples. The clarified saliva samples were passed through the 0.22-micron syringe filter to avoid any large impurities, and then mixed with 14% PEG (polyethylene glycol) vortexed, and incubated for 6–8 h at 4 °C. Furthermore, the mixture was subjected to centrifugation at 13,000 g for 1 h at 4 °C, and the pellet obtained was washed by adding 100 µl of 1 × PBS buffer to the sEVs pellet and decanting the PBS solution. This process was repeated twice and the washed pellet was re-suspended in 1 × phosphate buffer saline (PBS) [25, 26]. Furthermore, the obtained suspension was filtered using 100 kDa cut-off centrifugal filters. The ultrafiltration was performed after precipitation. The centrifugal filters were washed with 1 × phosphate buffer saline (PBS) before usage. The obtained suspension was loaded in the column and spun at 10,000 g to remove any small-size proteins and chemical precipitation contaminants. The sample retained in the filter is used for further experimentation. Chemical-based precipitation method was used to obtain a high yield of sEV, and the two-step filtration process provides purity and homogeneity.

### Morphological characterization by transmission electron microscopy

The ultrastructural morphology of isolated sEV was studied by transmission electron microscopy. The obtained sEV pellet was diluted with 0.1 M phosphate buffer (pH 7.4). The dilution ratio between controls and PD patients was 1:2. In brief, the isolated sEV were adsorbed on a 300-mesh carbon-coated copper grid (01843, Ted Pella) for 30 min at room temperature. The grids were blot dried and stained with 2% aqueous uranyl acetate solution for 10 s. Afterward, grids were blot dried and observed under the transmission electron microscope (Talos S, Thermo Scientific, USA).

### Nanoparticle tracking analysis

Nanoparticle tracking analysis aids in the determination of the concentration and the size of the particles in the nanometer range. The quantification of sEV was performed using the Nanoparticle Tracking Analysis (NTA) system from Particle Metrix, Germany, at different wavelengths. The concentration of sEV was determined in particle/ml in two modes of NTA: scatter mode, fluorescence mode: (lipid-binding dye-labeled), and antibody-labeled NTA. Both the methods, the dye-labeled mode of NTA, and the antibody mode of NTA work on the principle of the fluorescent mode of NTA. In the case of the fluorescence mode of NTA (dye-labeled), we used a laser of 640 nm for excitation, with an emission filter of 640 nm, whereas in the case of the antibody-based mode of NTA, we used a laser of excitation 488 nm, with an emission filter of 540 nm. The range of particle/frame was 50–500 after sample dilution, and a minimum of 500 number of traced particles was set for all experiments. The control experiments were performed including PBS buffer, fluorescence dye, and fluorescence antibody controls prior to the analysis of sEVs.

### Scatter-based mode of NTA

NTA aids in analyses (size, concentration) of the particle concentration in solution. All salivary isolated sEV samples were measured by NTA at 1:1000 dilutions in 1X-PBS buffer. Briefly, approximately 0.5 ml of diluted sEV sample was loaded into the sample chamber of the Zeta View Twin system. Three cycles were performed by scanning 11 cell positions each and capturing 60 frames per position (video setting: high) under the following settings: focus: autofocus; camera sensitivity for all samples: 80.0; shutter: 150; scattering intensity: 5.0; embedded laser at 488 nm; cell temperature: 25 °C. The videos were captured using a CMOS camera and analyzed by the in-build ZetaView Software 8.05.12 with specific analysis parameters: maximum particle size: 1000, minimum particle size 10, minimum particle brightness: 30.

### Fluorescence-based mode of NTA (dye-labeled mode of NTA)

In the fluorescence mode of NTA, the dye binds specifically to the lipid bilayer of the plasma membrane of the isolated sEV. For fluorescence-based NTA, isolated salivary sEV were incubated in the dilution of 1:1000 with the cell mask plasma membrane deep red (CMDR) stain (C10046, Invitrogen; stock concentration 5 mg/ml) for 3 h at room temperature. We used CellMask™ Deep Red Plasma Membrane Stain (C10046, Invitrogen) to label the small extracellular vesicles that are composed of the bilayer lipid membrane and express the surface ligands and receptors from its origin cells. The sEVs bilayer lipid membrane surrounds and contains a hydrophilic core; hence, the CellMask Deep Red dye binds specifically to the bilayer lipid membrane of the sEV without the cell-type differences. The CMDR stain has an excitation of 649 nm with an emission range of 666 nm. The excitation laser used is 640 nm, and the emission filter used is 660 nm. Briefly, approximately 0.5 ml of diluted sEV sample was loaded into the sample chamber of the Zeta View Twin system. The sEV-dye incubated sample was injected in the ratio of 1:500 dilution with the 1X-phosphate buffer saline. The lipid-dye bound sEVs were analyzed in the fluorescence mode of the Zeta View Twin system through a laser of 640 nm. Three cycles were performed by scanning 11 cell positions each and capturing 30 frames per position (video setting: high) under the following settings: focus: autofocus; camera sensitivity for all samples: 90.0; shutter: 200; scattering intensity: 3.0; embedded laser at 640 nm; cell temperature: 25 °C. The videos were captured using a CMOS camera and analyzed by the in-built ZetaView Software 8.05.12 with specific analysis parameters: maximum particle size: 1000, minimum particle size 10, minimum particle brightness: 30.

### Antibody-labeled mode of NTA

In the antibody-labeled NTA, the isolated salivary sEV were labeled with CD63 (a common tetraspanin for sEV). Only the extracellular vesicles that are CD63-positive will be observed in this process. The excitation of the primary conjugated CD63 antibody is 488 nm, and the emission (515–545) nm. The emission filter used is 540 nm, provided by the Particle Metrix, Germany. The CD63 antibody is specifically known to bind small-extracellular vesicles. For antibody-labeled NTA, sEV were incubated with 0.05 mg/ml CD63-Alexa 488 (IC5048G, R&D Systems; stock concentration 0.2 mg/ml) with an antibody to sample ratio of 1:10 for 2 h at room temperature. Briefly, approximately 0.5 ml of diluted sEV sample was loaded into the sample chamber of the Zeta View Twin system. The antibody-labeled sEVs were diluted in the ratio of 1:200 with the 1X-phosphate buffer saline before injecting into the system. Antibody-labeled sEV were analyzed in the fluorescence mode of the Zeta View Twin system through a laser of 488 nm. Three cycles were performed by scanning 11 cell positions each and capturing 30 frames per position (video setting: high) under the following settings: focus: autofocus; camera sensitivity for all samples: 95.0; shutter: 120; scattering intensity: 5.0; embedded laser at 488 nm; cell temperature: 25 °C. The videos were captured using a CMOS camera and analyzed by the in-built ZetaView Software 8.05.12 with specific analysis parameters: maximum particle size: 1000, minimum particle size 10, minimum particle brightness: 30.

### Western blot profiling

For Western blotting, 5 μl of sEV samples (each of isolated sEVs from control and patients in PBS) was used for volume normalization [27–29] (as mentioned, the starting sample volume was equal volume, i.e., 180 μl), were mixed with the sample loading dye and run on an 8–12% SDS PAGE. The obtained gel was subjected to wet-mode Western blotting using the BioRad Western blotting apparatus. The proteins were transferred from gel to 0.22 μm PVDF membrane followed by blocking using 3% BSA in Tris-base saline containing 0.1% of Tween 20 (TBST). The overnight incubation with the primary antibody (1:5000 dilution of antibody in 1.5% BSA in TBST) anti-CD9 (PA5-86,534, Invitrogen), anti-CD63 (10628D, Invitrogen), anti-Flotillin-1 (PA5-17,127, Invitrogen), anti-L1CAM/CD171 (MA1-46,045, Invitrogen), and anti-GAPDH (MA5-15,738, Invitrogen) was performed. The differential expression of the anti-phospho-α-syn (PA5-37,740, Invitrogen) was observed by running the 12% native PAGE [26]. The blot was developed using HRP-based electroluminescence using the Femto LUCENT™ PLUS-HRP kit (AD0023, Gbiosciences). As aggregated α-Synuclein (α-syn) is a key protein in Parkinson’s disease (PD), its phosphorylated form, pS129, has been found higher in PD patients than in healthy controls. To detect the expression of both forms of alpha-synuclein two antibodies were used. Total alpha-synuclein will detect the expression of all forms of alpha-synuclein proteins, for which ELISA was performed. However, the anti-phospho-alpha-synuclein antibody will only detect the alpha-synuclein protein which is phosphorylated at S129 amino acid, for which the Western blotting was performed. For concentration normalization, total protein concentration was determined using the bicinchoninic acid (BCA) protein assay kit (22,802, Pierce ThermoFisher Scientific) using bovine serum albumin (BSA) as standard to observe the total sEV protein concentration. An equal concentration of protein (2.5 µg) was loaded for CD9, sEV surface marker, from controls and patients.

### Enzyme-linked Immunosorbent Assay (ELISA)

Isolated sEV suspension was subjected to ultrasonication at an amplitude of 25 for 2 min with 30 s on and off-cycle. The sEV samples were subjected to freeze–thaw cycles prior to high amplitude sonication for efficient lysis of the sEVs. Furthermore, they were subjected to centrifugation at 10,000 g for 10 min, and the obtained supernatant was further utilized for experiments. Before loading the sEV samples into the ELISA plates, the sEV samples were at 37 °C for 15 min. Total protein concentration was determined using the bicinchoninic acid (BCA) protein assay kit (22,802, Pierce ThermoFisher Scientific) using bovine serum albumin (BSA) as standard. A total of 100 μg of quantified protein from sEV was screened for the presence of total alpha-synuclein protein using the ELISA kit (Human SNCα kit, E-EL-H0983, Elabsciences). All steps were followed according to the manufacturer’s protocol.

### Dopamine-associated transporter scan

^99m^Tc-TRODAT-SPECT/CT was performed for *n* = 41 subjects: 23 normal age-matched controls and 18 patients who were clinically diagnosed with Parkinson’s disease. The imaging of dopamine transporters (DAT) with Technetium-99 m (^99m^Tc)-labeled TRODAT-1([2-[[2-[[[3-(4-chlorophenyl)-8-methyl-8-azabicyclo[3,2,1]oct-2-yl]-methyl](2-mercaptoethyl)amino]ethyl]amino]ethanethiolato(3-)-N2,N2,S2,S2]oxo-[1R(exo-exo)]) single-photon emission tomography (SPECT)/CT was performed by nuclear medicine specialists. The scan was performed following the standardized protocol. For the scan, patients were requested not to administer dopaminergic drugs 6 h before the study.

### Image acquisition and reconstruction

The obtained raw data of the scan was scrutinized for any possible errors like head tilt and image quality at optima. The scans were evaluated for their picture quality, images with optimum quality, subject positioning, and registration for CT were included in the final analysis. For the generation of the composite slice, 8 transverse slices parallel to the canthomeatal line, and the slice portraying the highest activity of basal ganglia were taken. Extreme dorsal and ventral slices were not included to avoid errors by the partial volume effect. The images with positioning and head tilt, sub-optimal image counts, and CT coregistration issues were excluded.

### Quantification of binding of Technetium-99 m in TRODAT-SPECT/CT

Binding ratios of the Technetium-99 m in the whole striatum, caudate, and putamen with occipital cortex were determined by the image analysis of the DICOM studies of ^99m^Tc TRODAT-SPECT/CT. All images were reconstructed and displayed as axial sections. The SPECT images were co-registered with each patient’s corresponding CT images. A common platform, Osirix, was used for drawing regions of interest (ROIs) on bilateral whole striatum (WS), putamen (P), and caudate (C) on eight consecutive CT slices with the highest uptake in the striatum on corresponding SPECT slices. Binding ratios (BR) for bilateral caudate, putamen, and whole striatum were calculated with the occipital cortex (OC) as background. The region of OC portrays a comparatively low density of dopamine-associated transporters and hence was taken as a reference region or background. The uptake in each hemisphere of basal ganglia was calculated as count per pixel (CPP). The calculations of striatal binding ratios are undermentioned:

- Whole striatum binding ratios [WSBR]: (CPP whole striatum-CPP occipital cortex)/CPP occipital cortex
- Caudate binding ratios [CBR]: (CPP caudate nucleus-CPP occipital cortex)/CPP occipital cortex
- Putamen binding ratio [PBR]: (CPP putamen-CPP occipital cortex)/CPP occipital cortex

### Statistical analysis

The descriptive statistical analysis was used to determine the mean values of age, UPDRS score, H&Y score range, and disease duration. All the characterization and validation experiments: NTA, densitometric analysis of Western blotting, and ELISA, and the comparison of age-matched healthy controls and Parkinson’s disease patients were analyzed using GraphPad Prism 8.0 software. Statistical significance was calculated using the unpaired student t-test. Significance was considered at *p* < 0.05. Correlation between the fluorescence-tagged sEV concentration and alpha-synuclein as well as the binding ratios and NTA quantification was calculated using Pearson and Spearman’s rank correlation, respectively. During the statistical analysis of the data, we observed that the data was not under normal distribution, due to various factors like low sample size and data outliers. Hence, we have opted for non-parametric tests. We have incorporated the median value and the interquartile range in the figure legend. ROC is a probability curve that analyzes the accuracy of a test, and the full area under the ROC curve, AUC, represents the measure of separability. It tells how much the test is capable of distinguishing between classes. The higher the AUC, the better the test is at distinguishing between patients with the disease and those without the disease. The ROC curve is plotted with TPR (sensitivity) against the FPR (1-specificity) where TPR is on the *y*-axis and FPR is on the *x*-axis. An excellent test will have an AUC near 1 which means it has a good measure of separability. A poor test will have an AUC near 0 which means it has the worst measure of separability. The ROC analyses were performed to determine the accuracy of the fluorescence-labeled sEV assay. A non-parametric test Kruskal–Wallis test with a post hoc Dunn for three or more groups of data. The comparisons between the PD patient, prodromal PD, and HC were obtained using the Kruskal–Wallis test with Dunn’s multiple comparisons. All statistical tests used are one-sided.

## Results

There were mainly three categories of subjects included in the study, Parkinson’s disease patients (PD), healthy controls (HC), and prodromal cases. The healthy controls were in the age range of 40 to 75 years, the PD patients were in the age range of 30 to 79 years, and the prodromal cases were in the age range of 52 to 75 years. The age range of the subjects is given in column 1 of Table 1. The mean age of patients, healthy controls, and prodromal were 56.20 years, 55.0 years, and 58.25 years, respectively. The mean values of the UPDRS score, H&Y score, and the duration of the disease are mentioned in Table 1. All values are given with the standard deviation.

**Table 1:** Demographic details and mean values of the scoring assessment as well as disease duration

All samples	Age (range in years)	UPDRS (range)	H&Y (range)	Disease duration (years)
Healthy controls (26)	40–75	NA	NA	NA
Prodromal cases (08)	52–75	NA	NA	NA
Parkinson’s disease (70)	30–79	84.21 ± 5.106 (1–183)	1.952 ± 0.1039 (0–4)	6.790 ± 0.5248 (1.5–20.0)

### Characterization and validation of sEVs

**Fig. 1:**
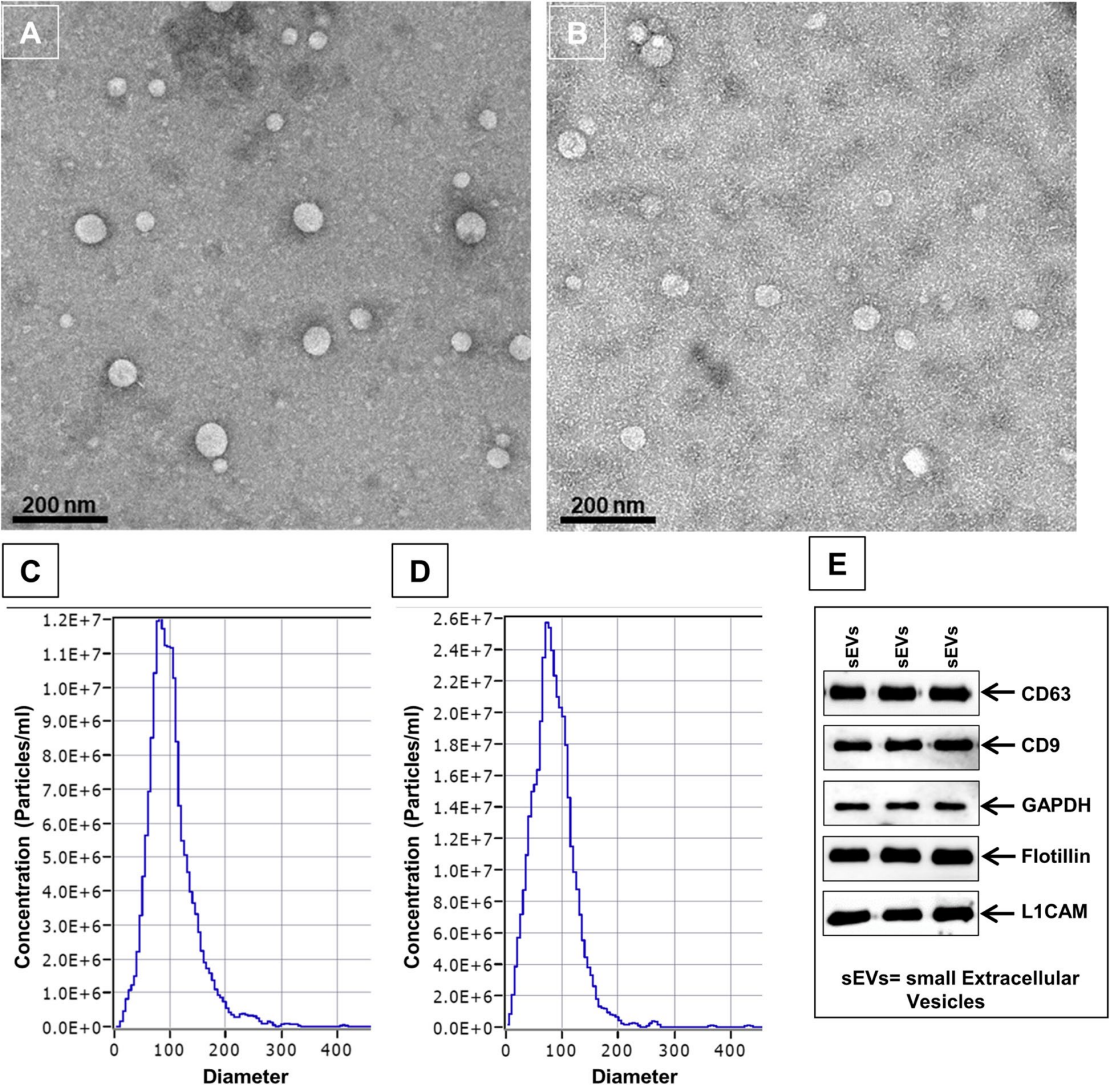
Characterization and validation of isolated salivary sEVs. Morphological characterization of isolated salivary sEV through transmission electron microscopy (scale bar 200 nm) from age-matched healthy controls (**A**) and PD patients (**B**) in a ratio of 1:2 dilution. Graphical representation of distribution of size of sEV sub-populations (nm) vs concentration (particle/ml) in age-matched healthy controls (**C**) and PD patients (**D**) (dilution factor: 1000 × in both). **E** Western-blot of CD63, CD9, Flotillin-1, L1CAM, and GAPDH for sEV validation

The isolated sEV from the saliva samples of subjects are characterized and validated using the MISEV 2018 criteria established to work specifically with extracellular vesicles [30]. According to the MISEV 2018 criteria to analyze the purity and demonstrate the efficient isolation of small extracellular vesicles, we used the protein characterization markers from categories 1, 2, and 3 given in Table 3 of the MISEV criteria [30]. The isolated sEVs have been reported as the heterogeneous population in clinical samples, therefore, we used volume normalization for all experiments using an equal volume of starting samples, i.e., saliva in all groups, as well as an equal volume of isolated sEVs in each experiment. The morphological characterization of sEVs was performed through transmission electron microscopy, as shown in Fig. 1A and B. The representative TEM images of the isolated salivary sEVs are shown on a scale of 200 nm. The sEV appears round/spherical with the outer lipid bilayer. The two steps filtration process described in the methods aided in achieving the pure and largely homogeneous population of small extracellular vesicles (Fig. 1A and B, Additional file 1: Fig. S1). We observed a higher sEVs population in the PD than in the healthy control. The representative image of the scatter-mode NTA (dilution factor of 1000 × in both controls as well as PD patients) shows the mean size of the isolated salivary sEV in Fig. 1C and D, which displays the size distribution of all subpopulations of the sEV. The graphical representation is made between the concentrations of sEV in particle/ml with the diameter of particles in the nanometer range. The average size appeared to be around 100 nm, constituting 98% of the sample. For the validation of the isolated sEV, Western blotting was performed using the surface marker CD63, CD9, cytosolic side membrane marker Flotillin-1, the cytosolic marker GAPDH, and the confirmation of the neuron-associated protein CD171/L1CAM (Fig. 1E).

### NTA-based determination of sEV concentration between HC and PD patients

**Fig. 2:**
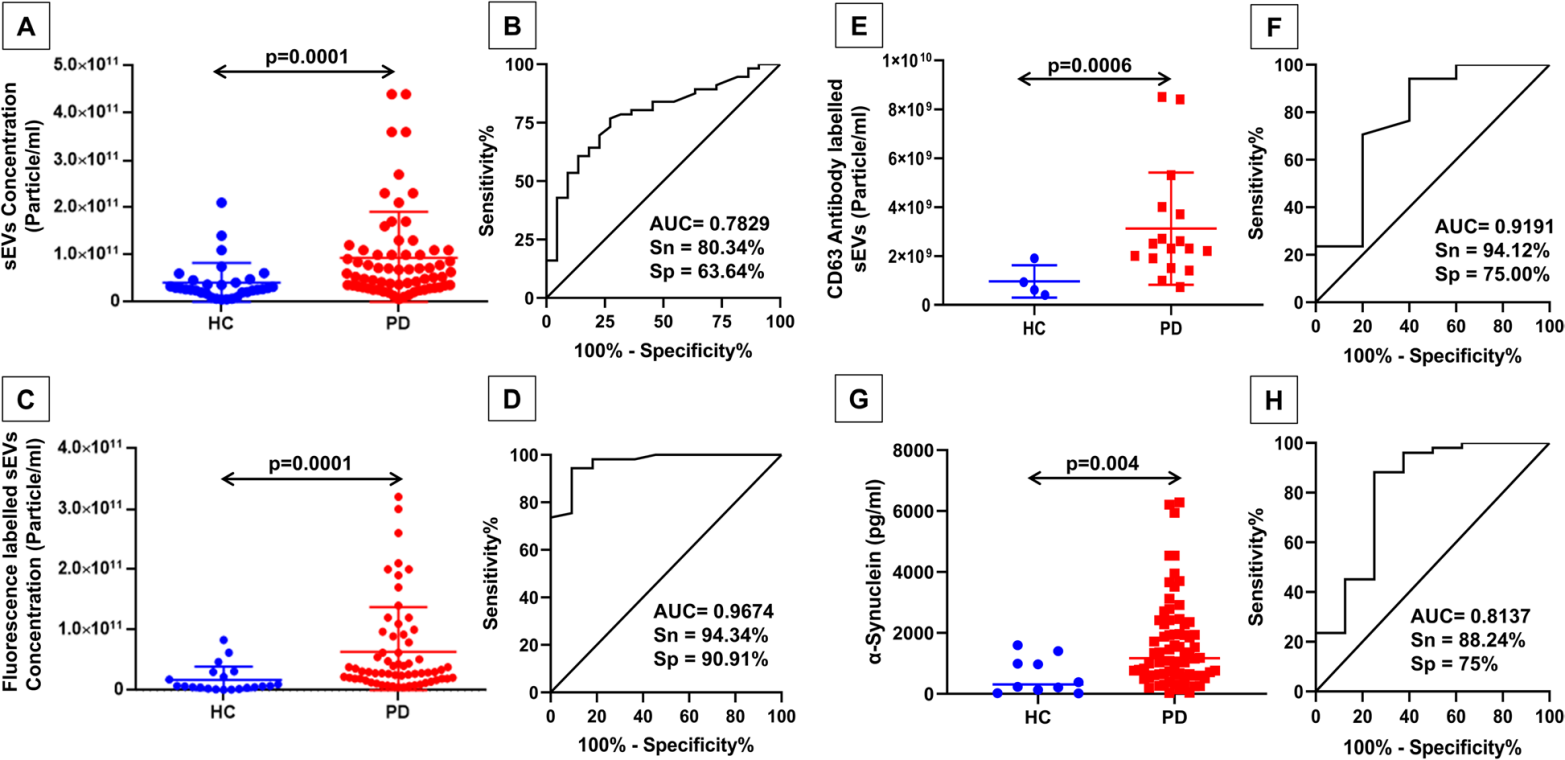
Salivary sEVs concentrations in age-matched healthy controls and PD patients. **A** Salivary sEV levels (particle/ml) from healthy controls (HC) and PD patients in scatter mode NTA (*p* = 0.0001) and its ROC curve analysis (**B**). **C** Fluorescence-dye-labeled salivary sEV levels (particle/ml) from HC and PD patients in fluorescence mode NTA (*p* = 0.0001) and its ROC curve analysis (**D**). **E** CD63 protein-specific salivary sEV levels (particle/ml) from HC and PD patients in antibody mode NTA (*p* = 0.0006) and its ROC curve analysis (**F**). **G** Alpha-synuclein protein levels (pg/ml) in the salivary sEV from HC and PD patients (0.004) by ELISA and its ROC curve analysis (**H**). All graphs are presented with mean ± SEM

The quantification of sEV was performed using the scatter and fluorescence mode of the NTA. The measurement of sEV concentration in scatter mode of NTA showed that the concentration of sEV (particle/ml) was higher in PD patients compared to HC (*p* = 0.0001), as shown in Fig. 2A. The mean concentrations of sEV measured by all NTA modes in scatter mode NTA of PD patients and controls are tabulated form in Table 2. The average size appeared to be around 100 nm range particles (controls 90.15 ± 13.15 nm, and PD 105 ± 18.67 nm), constituting 98% of the sample. The ROC and AUC analyses were performed for scatter mode NTA, and the cut-off value for sEV concentration was calculated to differentiate PD patients from healthy controls. The ROC curve analysis has an AUC = 0.7829, with a sensitivity of 80.34% and specificity of 63.64% (Fig. 2B). The fluorescence mode of NTA was performed using the lipid-binding cell-mask deep red plasma membrane stain. The findings of the fluorescence-dye mode of NTA substantiate the findings of the scatter mode of NTA with great sensitivity and specificity.

**Table 2:** Mean values in scatter, fluorescence, and antibody-based mode of nanoparticle tracking analysis

**Subjects**	**Scatter mode** Mean concentration (particle/ml)	**Fluorescence mode** Mean concentration (particle/ml)	**Antibody mode** Mean concentration (particle/ml)
Parkinson’s disease patients	8.057E + 10	6.2E + 10	3.119E + 9
Healthy age-matched controls	4.292E + 10	1.734E + 10	6.635E + 8

In particular, the fluorescence-dye-labeled sEV concentration was very high in PD compared to HC (*p* = 0.0001), shown in Fig. 2C. The ROC curve analysis to distinguish the HC from PD has shown AUC = 0.9674, with a sensitivity and specificity of 94.34% and 90.91% (Fig. 2D), respectively. To validate the results obtained through fluorescence mode, we performed antibody-based NTA. The CD63 Alexa fluor 488 antibody-labeled sEV concentration was observed to be higher in PD compared to HC (*p* = 0.0006), as shown in Fig. 2E. The ROC analysis reported the AUC value relative to the antibody-tagged sEV (AUC = 0.9191) with a sensitivity of 94.12% and specificity of 75% (Fig. 2F). Based on these results, we can predict that the fluorescence mode of NTA has shown a high AUC value with high sensitivity and specificity, thus providing the proof of concept for consideration of the fluorescence mode of NTA in the diagnosis of early-stage PD. Furthermore, we confirmed these findings with α-syn ELISA in controls as well as PD patients (Fig. 2G and H), which is explained in the ELISA section of the results.

### Differential expression of proteins in the sEV of healthy controls and PD patients through antibody profiling

**Fig. 3:**
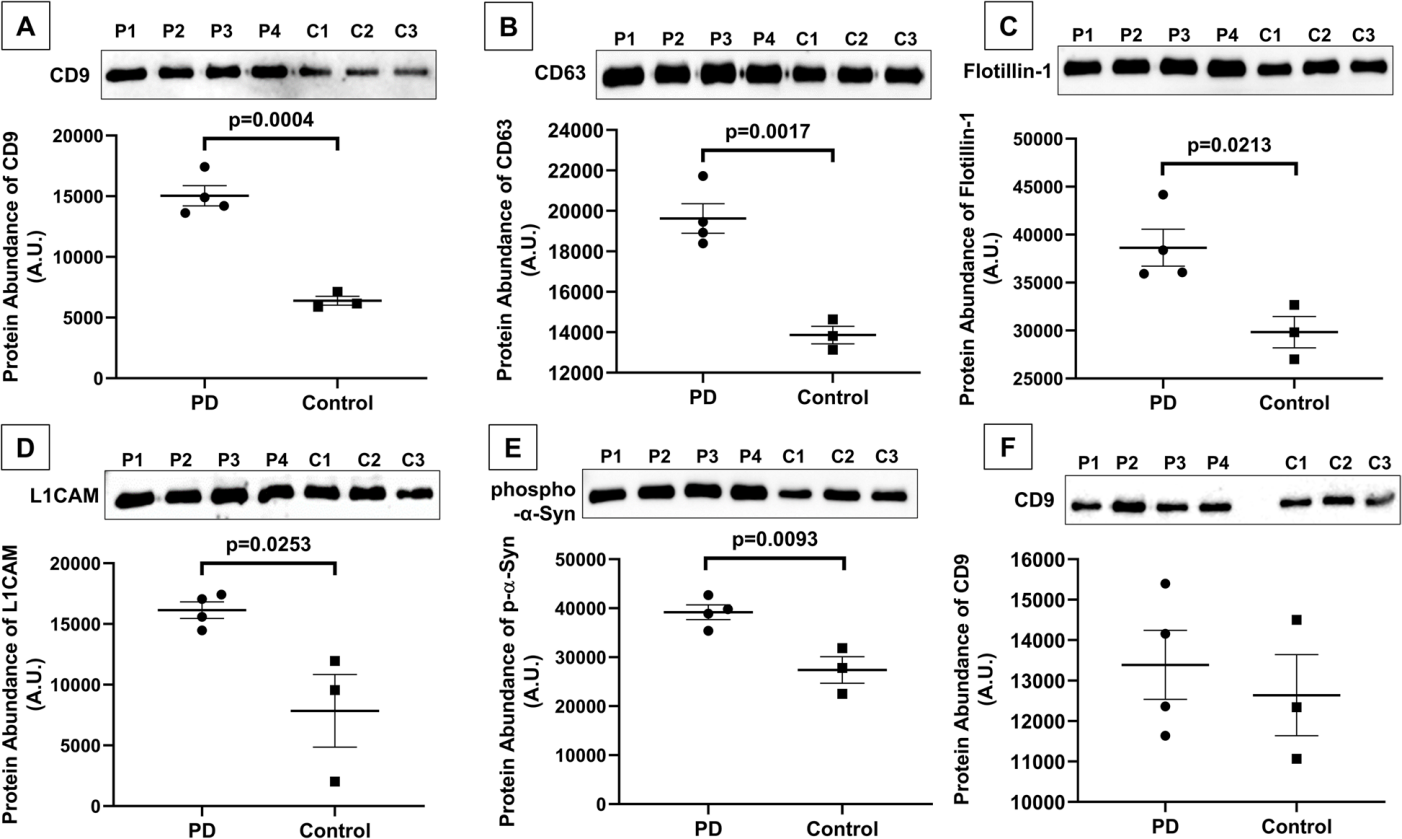
Differential expression of proteins in the sEV in PD patients (P1–P4) and healthy controls (C1–C3). **A** CD9 as sEV surface marker of PD and HC and its densitometric analysis (*p* = 0.0004). **B** CD63 as sEV surface marker of PD and HC and its densitometric analysis (*p* = 0.0017). **C** Flotillin-1 from sEV of PD and HC and its densitometric analysis (*p* = 0.0213). **D** L1CAM, a neuronal protein marker of PD and HC, and its densitometric analysis (*p* = 0.0253). **E** Phospho-alpha-synuclein from sEV of PD and HC and its densitometric analysis (*p* = 0.0093). **F** CD9 as sEV surface marker of PD and HC and its densitometric analysis, with the protein concentration normalization. All graphs are presented with mean ± SEM

The isolated sEV from the subjects’ saliva was validated using Western blotting. The densitometry of the band intensities of each independent sample in all the Western blots was done using the ImageJ software. The purity of isolated sEVs was observed in a Western blot using CD63 (surface marker). CD63 expression was observed in the isolated sEVs pellet but was not observed in the supernatant (Additional file 1: Fig. S1) obtained during the isolation process. To observe the differences between PD patients and controls, Western blot volume normalization was used where we loaded an equal sample volume (5 µl) from PD and healthy controls (similarly done in the NTA); and here, we observed a significant increase in CD9 (*p* = 0.0004) (Fig. 3A, Additional file 1: Fig. S2) in PD patients than healthy controls. We observed these changes due to the concentration (number) of sEV varying between PD and healthy control, which is also reflected in the expression of loading control in a similar trend. Similarly, in a Western blot of CD63, the expression was significantly increased in PD patients than in healthy controls (*p* = 0.0017) (Fig. 3B, Additional file 1: Fig. S3). Another marker for the validation used was Flotillin-1, a cytosolic marker for sEV, shown a higher expression in PD patients than in controls (*p* = 0.0213) (Fig. 3C, Additional file 1: Fig. S4). The neuronal origin of the isolated salivary sEV was confirmed by Western blotting against anti-L1CAM/CD171 antibody which is similarly increased in PD patients than controls (*p* = 0.0253) (Fig. 3D, Additional file 1: Fig. S5). This neuronal protein (L1CAM) is present in the soluble form and on the surface of neuronal-derived sEV. In addition, the differential expression of the PD biomarker protein phospho-α-synuclein as the sEV cargo protein was observed through the Western blot and found to be higher in PD patients than controls (*p* = 0.0093) (Fig. 3E, Additional file 1: Fig. S6). The expression profile of loading control, GAPDH, was observed in similar trends with corresponding protein markers (Additional file 1: Figs. S2–S6). We further did the Western blot with concentration normalization as a reference to substantiate that both the groups (PD and HC) should not have differences in the numbers of sEV (Fig. 3F, Additional file 1: Fig. S7). We observed a similar intensity band in the Western blot in both groups.

### Quantification of total α-syn by ELISA and correlation between α-syn and sEV concentration

**Fig. 4:**
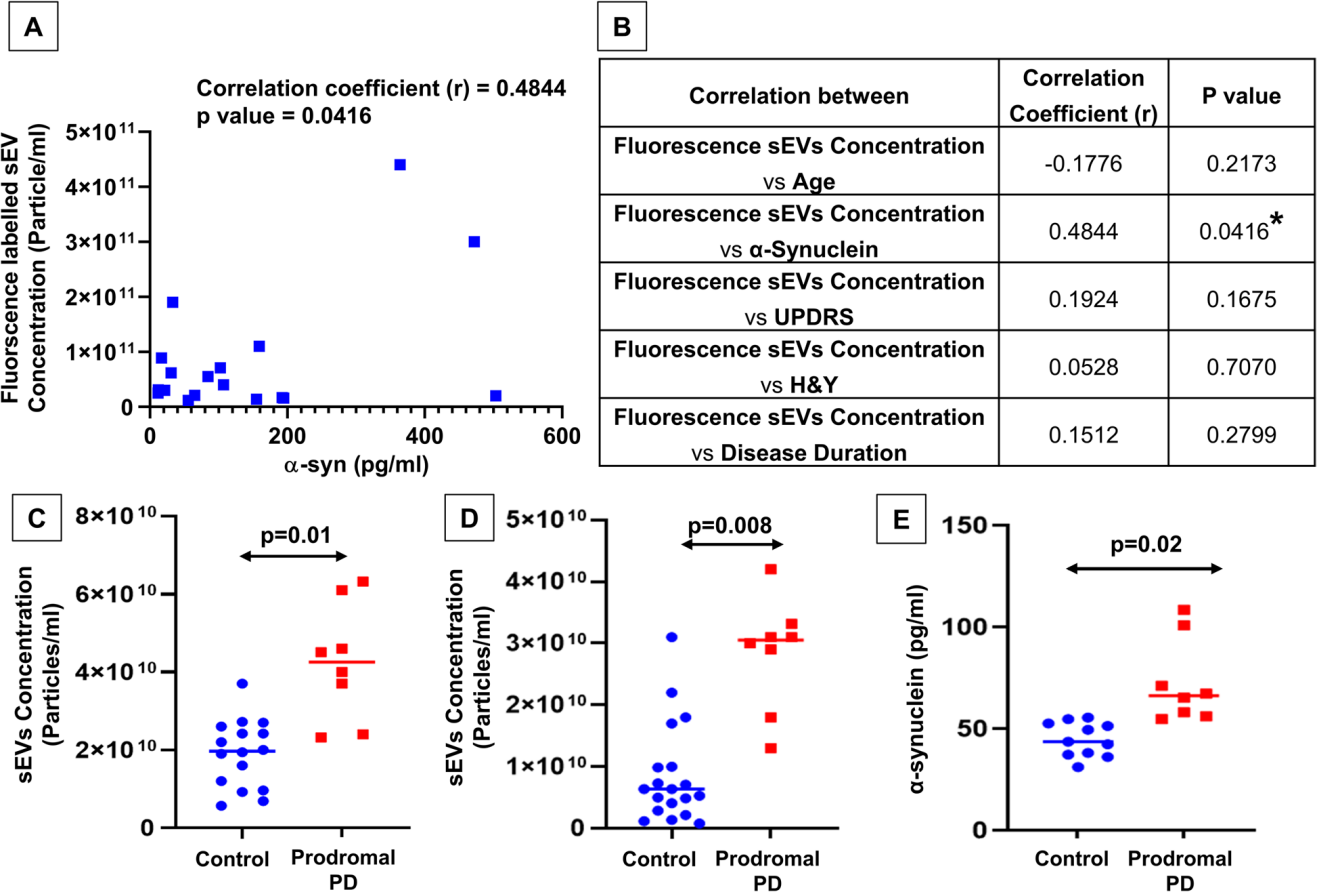
Fluorescence-dye-labeled sEV. **A** Correlation coefficient analysis between fluorescence-dye-labeled sEV concentration with α-synuclein protein levels (*p* = 0.0416), “*r*” represents the correlation coefficient. **B** Tabular representation of the correlation between the fluorescence-dye-labeled sEV concentration and age, α-synuclein protein levels, UPDRS scores, H&Y scores, and duration of the disease. Quantification of salivary sEV concentration of prodromal PD and HC: salivary sEV levels from HC and prodromal PD are represented in NTA of scatter mode (*p* = 0.01) (**C**); and fluorescence-dye mode (*p* = 0.008) (**D**). Similarly, **E** alpha-synuclein protein determination from sEV of HC and prodromal PD cases (*p* = 0.02). All graphs are presented with mean ± SEM

A sandwich ELISA experiment was performed to quantify the total α-synuclein protein in the small extracellular vesicular cargo of all subjects. To calculate the final concentration, we multiplied the OD of each subject with a dilution factor of 10 followed by the calculation of the concentration using the equation *y* = *m* × *x* + *c*, and in our experiment, the equation is *y* = 0.0024 × *x* + 0.1321. ELISA outcome suggested a higher expression of the total α-synuclein protein in PD patients compared to HC (*p* = 0.004), which corroborated with the results of NTA (Fig. 2G and H). The ROC analysis between PD patients and HC revealed that the AUC value was 0.8137, with 88.24% and 75% sensitivity and specificity, respectively. Based on the fluorescence of sEV concentration ROC data, we can say that it provides a better cut-off value for differentiating between HC and PD patients. Furthermore, a correlation coefficient analysis between fluorescence sEV concentration and various parameters was performed (Fig. 4A and B). The correlation between the fluorescence sEV concentration (dye-labeled NTA) and the α-synuclein protein concentration (pg/ml) showed a positive correlation (*r* = 0.4844) with a significant *p*-value (*p* = 0.0416). We also performed normalization of total alpha-synuclein cargo per EV. In healthy controls, the total alpha-synuclein cargo per sEV particle was determined to be around 170.52 × 10^−10^ pg total alpha-synuclein per particle. In contrast, PD patients exhibited a higher value of around 200.32 × 10^−10^ total alpha-synuclein per particle, indicating an elevated total alpha-synuclein cargo per sEV in PD patients compared to healthy controls. Furthermore, we have calculated the ratio of phospho-alpha-synuclein/total alpha-synuclein which was found to be significantly high in PD patients as compared to healthy control (*p* = 0.003). This analysis established that PD patients with increased sEV concentration also exhibited higher levels of alpha-synuclein expression, further substantiating our results.

### Determine the cases of prodromal PD

Based on our analysis, we were speculating that some of the recruited healthy controls could be cases of prodromal Parkinson’s disease (Fig. 4). The subjective assessment test (UPDRS) is used to observe the progression and severity of PD and does not apply to disease recognition. In the cases of prodromal PD, the scoring was a little higher but well within the range of the healthy controls. The subjects of prodromal PD qualified as healthy controls based on the neuropsychological assessments, and have shown different molecular changes. For a better understanding, we assumed these subjects could be in the asymptomatic stages of the disease, and the underlying reasons for our assumptions are the increased concentration of sEV (particle/ml) in prodromal PD compared to HC (*p* = 0.01) (Fig. 4C, Additional file 1: Fig. S8). We observed a better trend with the fluorescent-dye-tagged NTA (*p* = 0.008) (Fig. 4D). Similarly, the α-syn protein concentrations obtained through ELISA were also significantly increased in prodromal PD (*p* = 0.02) in comparison to HC, shown in Fig. 4E. Conclusively, we assumed that these subjects could be prodromal Parkinson's disease cases.

### Quantification of the Technetium-99 m binding

**Fig. 5:**
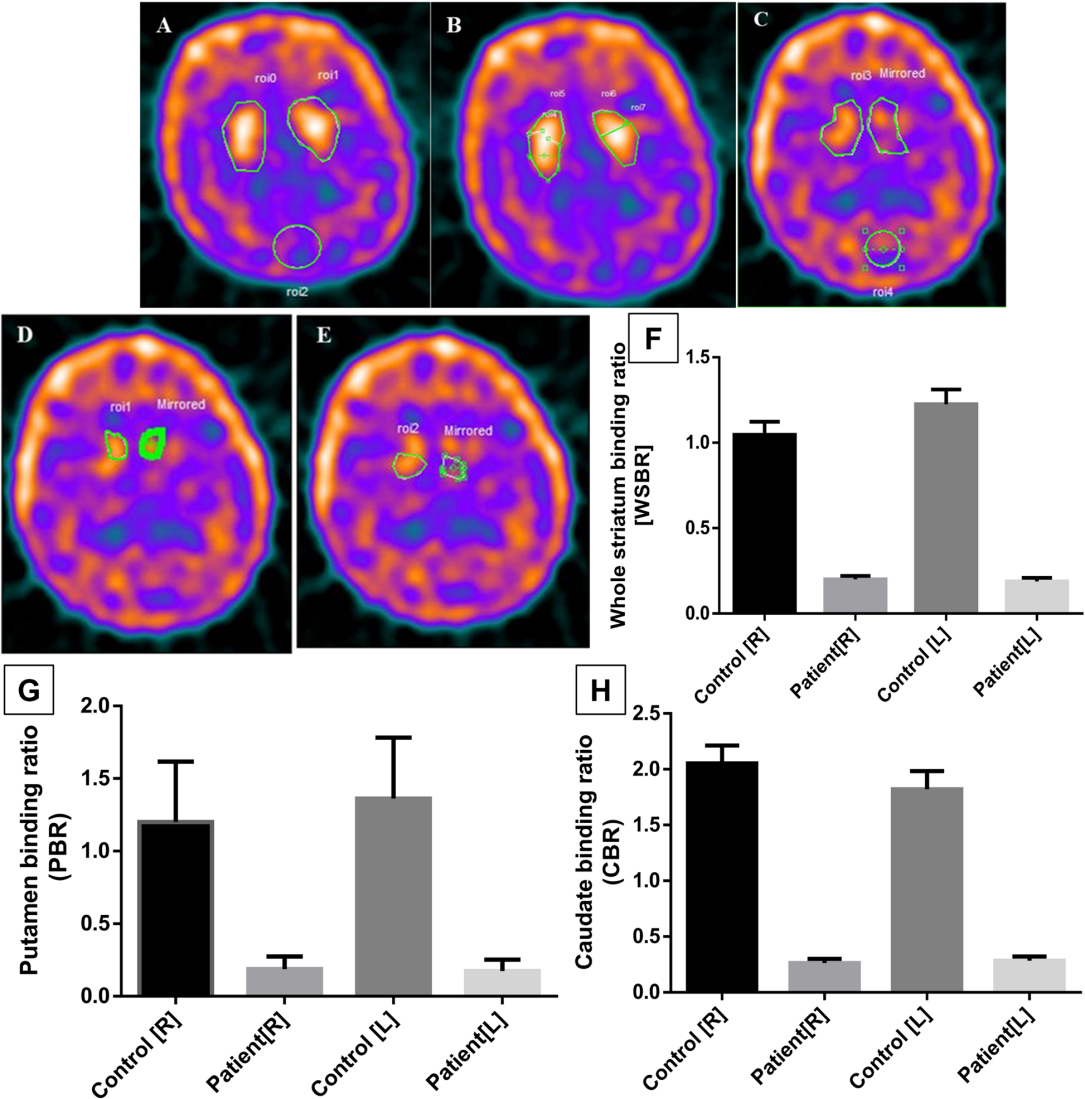
^99m^Tc TRODAT-SPECT imaging of healthy age-matched control and Parkinson’s disease patients. **A**, **B** Representative images for the basal ganglia of the healthy age-matched control with the typical striatal region of interests (ROIs): **A** whole striatum (upper marked areas) and occipital (lower circle), **B** left and right caudate (upper marked areas) and left and right putamen (lower marked areas). **C**, **D**, and **E** Representative image for the basal ganglia of Parkinson’s disease patients with a typical striatal region of interest (ROI): **C** whole striatum (upper marked areas) and occipital (lower circle), **D** left and right caudate, **E** left and right putamen. Comparison of the whole striatum binding ratio (**F**), putamen binding ratio (**G**), and caudate binding ratio (**H**) between PD and HC

The representative images of the HC and PD patients obtained from the DICOM studies are shown in Fig. 5A and B for the HC and Fig. 5C, D, and E for the PD patient. The composite slice was made from 8 transverse slices using Xeleris software. The ROIs are marked in the images and show the whole striatum, caudate, putamen, and occipital cortex regions. The binding ratios in the basal ganglia regions of Technetium-99 m are tabulated in Table 3, all ratios present as mean ± SD. The quantification of the ROIs marked in the representative images of the HC and PD patients was performed using the Osirix software and reported in ratios with occipital as the background. The binding ratios from the TRODAT-SPECT/CT were higher in healthy controls compared to PD, as shown in Table 3. The graphical representation of the striatal ratio differences between HC and PD patients is shown in Fig. 5F, G, and H. The Spearman rank analysis was performed between the WSBR, CBR, and PBR of the bilateral basal ganglia with the sEV concentration, we observed a positive correlation between the CBR [L] and sEV concentration (*r* = 0.8117) with *p* = 0.0112. Nevertheless, the caudate binding ratio positively correlates with the NTA-based measured sEV (scatter, fluorescence-labeled, and antibody-labeled) and the α-syn protein concentration.

**Table 3:** Comparison of quantitative values of ^99m^T_c_ uptake in TRODAT-SPECT. The uptake is in the whole striatum, putamen, caudate and occipital in the form of whole striatum binding ratios, putamen binding ratio, and caudate binding ratio between the Parkinson’s disease patient and healthy age-matched controls

Quantitative parameters	Parkinson’s disease patients (Mean ± SD)	Healthy age-matched controls (Mean ± SD)
**Whole striatum binding ratio [WSBR]**		
[WSBR]-right	0.1999 ± 0.0694	1.046 ± 0.3588
[WSBR]-left	0.2041 ± 0.099	1.226 ± 0.4041
**Putamen binding ratio [PBR]**		
[PBR]-right	0.1865 ± 0.084	1.200 ± 0.4180
[PBR]-left	0.1894 ± 0.0987	1.362 ± 0.4198
**Caudate binding ratio [CBR]**		
[CBR]-right	0.2605 ± 0.1353	2.054 ± 0.7458
[CBR]-left	0.3027 ± 0.1482	1.823 ± 0.7616

## Discussion

In this study, we aimed to design a method based on the fluorescence NTA of the salivary sEV to address the benefits of early diagnosis of PD. The role of sEV in the propagation of disease pathologies in neurodegenerative diseases and psychiatric diseases is well known [10, 17, 31, 32]. The specific trigger due to which the monomeric form of α-syn acquires the neurotoxic oligomeric form and makes the large aggregates that result in Lewy pathology is due to disturbances in the lysosomal autophagy system (LAS) that leads to inefficient clearance of α-syn oligomeric assemblies [33, 34]. The increased sEV secretion and the transfer of disease pathologies make them a potential candidate that can give a fingerprint of the molecular status of their originating cell. Our study attempts to explicate the fluorescence-tagged salivary sEV that has similar relationships with the hallmark protein (α-syn) for the diseases, and this can be used as an early diagnostic methodology in PD.

The key findings of this study revealed the increased salivary sEVs concentration in PD patients in all modes of NTA. The sEV were isolated using a rigorous isolation protocol combining the chemical precipitation method followed by ultrafiltration [30]. The sEV suspension from precipitation is followed by an ultrafiltration step that aids in concentrating the sEV suspension and removal of traces of precipitant and very small protein contaminants. We identified the purity and efficient isolation of sEV using CD63 (surface marker) expression in the sEV pellet and supernatant. We also used the neuronal protein L1CAM to check the neuronal origin, although the use of this marker for specific CNS-derived vesicles is contradicted [35]. Nevertheless, in our study, the neuron-related protein is used to check the protein markers and not for L1CAM affinity-based isolation. Two steps filtration procedure has accompanied the sEV isolation method in our study to ensure high purity.

Nonetheless, with the fluorescent-dye-labeled salivary sEVs, we achieved the sensitivity and specificity (AUC = 0.967, 94.34% sensitivity, 90.91% specificity) that can be clinically acceptable for a method. This fluorescent dye specifically binds to the lipid bilayer of the plasma membrane. Thus, distinctively it binds to the sEV in the nanometer range and effectively distinguishes them. To validate the fluorescence-tagged results, we worked on an Alexa fluor 488 conjugated anti-CD63 antibody on sEV. The CD63-antibody-labeled salivary sEV concentration supported the fluorescence-tagged results with similar accuracy (AUC of 0.9191, a sensitivity of 94.12%). sEVs have a heterogeneous population of the protein, and hence, not all sEVs carry similar markers; instead, the markers are changing depending on the sEV subset [35, 36]. Therefore, to characterize sEVs, different markers can be used to observe the overall population of sEVs [10, 30, 36]. To reduce the variations between results, we used different modes of NTA to characterize the heterogeneous population of sEVs in saliva samples from controls, prodromal, and PD. To further substantiate our findings, we evaluated the expression profiles of sEV markers (CD9, CD63, Flotillin-1), a neuronal marker (L1CAM), and PD specific phospho-α-synuclein in isolated sEVs from PD patients and healthy controls. In this study, PD patients show significantly increased expressions of sEV markers (CD9, *p* = 0.0004; CD63, *p* = 0.0017; Flotillin-1, *p* = 0.0213), a neuronal marker (L1CAM, *p* = 0.0253) and PD specific phospho-α-synuclein (*p* = 0.0093) than healthy controls due to higher numbers of sEV in PD patients as observed in the NTA experiments. It is also reflected in similar trends in loading control as housekeeping protein expression should also change with the number of sEV. Similarly, the differential expression of α-syn from the salivary sEV cargo detected by ELISA is significantly higher in PD than in HC.

The ROC curve analysis for the α-syn expression has an AUC of 0.8137 and a sensitivity of 88.24%. Our study's primary outcomes contradicted the total α-syn levels in the CSF [37, 38] but aligned with plasma and saliva α-syn levels [39] detected by immunoassays in the previously published studies. Nonetheless, the data from the meta-analysis of total CSF α-syn shows low diagnostic accuracy [37], whereas our approach resulted in a similar AUC of 0.96 in the case of the fluorescent dye-labeled salivary sEV. The outcome of our validation study on sEV cargo, α-syn_Total_ showed better sensitivity and specificity (AUC: 0.81, sensitivity: 88%, specificity: 75%) in comparison to the other work (AUC: 0.657, sensitivity:71.2%, specificity 50.0%) [38]. In establishing the correlation between the PD hallmark protein α-syn and the fluorescent dye-labeled salivary sEV, we observed a positive correlation of *r* = 0.4844 and a significant *p*-value (0.0416). Furthermore, we propose for the first time how the fluorescent-dye-labeled salivary sEV and α-syn are correlated. A study published by Cao et al.(2019) shows the level of salivary sEV α-syn_Olig_ and α-syn_Olig_/α-syn_Total_ in HC versus PD Western blot profiling and obtained the AUC = 0.941, 92% sensitivity, 86% specificity for α- syn_Olig_ as well the AUC = 0.772, 81% sensitivity and 71% specificity for α-syn_Olig_/α-syn_Total_ [40], whereas our study independently with the fluorescent-dye-labeled salivary sEV supported with the antibody (α-syn_Total_) has higher diagnostic accuracy. Although the antibody-based determination of sEV concentration is more specific to sEV-surface markers; however, it is expensive, and chances of variations occur due to several steps, whereas the fluorescence dye-based method is easy and cost-effective, henceforth proving to be more suitable for developing a possible robust detection protocol. Our group is also working on other neurological disorders, i.e., Alzheimer’s disease (AD), where we observed the changes in the sEV concentration in control, mild cognitive impairment (MCI), and AD [25]. For the confirmed diagnosis, the anatomical imaging (CT, MRI) does not show significant differences between PD and other PD-like conditions; therefore, the approaches like positron emission tomography (PET) and SPECT with the radio-labeled molecules that specifically bind with the target are found to be more efficient [41–44]. ^99m^Tc-TRODAT-SPECT/CT, in which the Technetium-99 mm binds with the dopamine transporter (DAT), is target-specific and serves as a representation of the density of dopaminergic neurons [45, 46]. In our study, we quantified the binding of Technetium-99 m to the DAT. The uptake ratios in the bilateral whole striatum, caudate, and putamen are higher in the healthy age-matched controls compared to PD, thus confirming the diagnosis of the patients. The differences between the binding ratios of HC and PD are mentioned in Table 3. Some of the earlier studies suggested the correlation between the striatal ratios and the α-syn_Total_ in the blood plasma [47] of the PD, and our results displaying the positive trend of the striatal ratios and α-syn_Total_ are in concordance with it.

## Conclusions

We found that some of the recruited healthy control samples could be cases of prodromal PD, as these subjects had higher concentrations of the fluorescent-dye-tagged salivary sEV and α-syn_Total_ compared to HC, but had lower concentrations in comparison to the PD samples, therefore, we assume that these subjects might be in the asymptomatic stages of Parkinson’s disease, although they showed no clinical symptoms for assessment through UPDRS examination. Nevertheless, this can show that the fluorescent-dye-tagged salivary sEV could be a carrier of the molecular signature of PD and has the potential to act as a susceptibility-risk biomarker.

### Supplementary Information

**Additional file 1:** **Fig. S1.** [Western blot of anti-CD63. In this blot, the pellet obtained during the sEV isolation method and the supernatant, show the purity of sEVs. *P*= Pellet obtained by the PEG-based precipitation combined with ultrafiltration. S= Supernatant obtained during the isolation process]. **Fig. S2.** [Expression profile of CD9 in PD patients (P1-P4) and healthy controls (C1-C3). (A) Western blot of anti-CD9 and anti-GAPDH with equal sample volume (5µl) in PD and HC. Densitometric analysis of (B) anti-CD9 (*p*=0.0004) and (C) anti-GAPDH (*p*=0.0137). GAPDH is a loading control. All graphs are presented with Mean ± SEM]. **Fig. S3.** [Expression profile of CD63 in PD patients (P1-P4) and healthy controls (C1-C3). (A) Western blot of anti-CD63 and anti-GAPDH with equal sample volume (5µl) in PD and HC. Densitometric analysis of (B) anti-CD63 (*p*=0.0017) and (C) anti-GAPDH (*p*=0.047). GAPDH is a loading control. All graphs are presented with Mean ± SEM]. **Fig. S4.** [Expression profile of Flotillin-1 in PD patients (P1-P4) and healthy controls (C1-C3). (A) Western blot of anti- Flotillin-1 and anti-GAPDH with equal sample volume (5µl) in PD and HC. Densitometric analysis of (B) anti-Flotillin-1 (*p*=0.0213) and (C) anti-GAPDH (*p*=0.0448). GAPDH is a loading control. All graphs are presented with Mean ± SEM]. **Fig. S5.** [Expression profile of L1CAM in PD patients (P1-P4) and healthy controls (C1-C3). (A) Western blot of anti-L1CAM and anti-GAPDH with equal sample volume (5µl) in PD and HC. Densitometric analysis of (B) anti-L1CAM (*p*=0.0253) and (C) anti-GAPDH (*p*=0.0399). GAPDH is a loading control. All graphs are presented with Mean ± SEM]. **Fig. S6**. [Expression profile of Phospho-α-Synuclein in PD patients (P1-P4) and healthy controls (C1-C3). (A) Western blot of anti-phospho-α-Synuclein and anti-GAPDH with equal sample volume (5µl) in PD and HC. Densitometric analysis of (B) anti- phospho-α-Synuclein (*p*=0.0093) and (C) anti-GAPDH. GAPDH is a loading control. All graphs are presented with Mean ± SEM]. **Fig. S7.** [Expression profile of CD9 with Concentration Normalization in PD patients (P1-P4) and healthy controls (C1-C3). (A) Western blot of anti-CD9 and anti-GAPDH with equal protein sample loading (2.5µg) in PD and HC. Densitometric analysis of (B) anti-CD9 and (C) anti-GAPDH. GAPDH is a loading control. We did not observe significant differences in CD9 and GAPDH expressions in PD and HC. All graphs are presented with Mean ± SEM]. **Fig. S8.** [Correlation coefficient analysis between sEV concentration and UPDRS score in controls and PD patients].

## Data Availability

All data generated or analyzed during this study are included in this published article and its supplementary information files. Further data information is available from the corresponding author on reasonable request.

## Abbreviations

99mTc TRODAT-SPECT scan: Technetium-99 m (99mTc)-labeled TRODAT-1([2-[[2-[[[3-(4-chlorophenyl)-8-methyl-8-azabicyclo[3,2,1]oct-2-yl]-methyl](2-mercaptoethyl)amino]ethyl]amino]ethanethiolato(3-)-N2,N2,S2,S2]oxo-[1R (exo-exo)]) single-photon emission computed tomography
BR: Binding ratios
MMSE: Mini-Mental State Examination
NTA: Nanoparticle tracking analysis
PD: Parkinson’s disease
sEV: Small extracellular vesicles
UPDRS: Unified Parkinson’s Disease Rating Scale
α-syn: Alpha-synuclein
