# Supplementary material for: Fluorescence-tagged salivary small extracellular vesicles as a nanotool in early diagnosis of Parkinson’s disease
Source: BMC Med. 2023 Sep 4;21:335. doi: 10.1186/s12916-023-03031-1 (PMC10478478; doi:10.1186/s12916-023-03031-1)
Supplement: Supplementary file 1 — Additional file 1: Fig. S1. [Western blot of anti-CD63. In this blot, the pellet obtained during the sEV isolation method and the supernatant, show the purity of sEVs. P= Pellet obtained by the PEG-based precipitation combined with ultrafiltration. S= Supernatant obtained during the isolation process]. Fig. S2. [Expression profile of CD9 in PD patients (P1-P4) and healthy controls (C1-C3). (A) Western blot of anti-CD9 and anti-GAPDH with equal sample volume (5µl) in PD and HC. Densitometric analysis of (B) anti-CD9 (p=0.0004) and (C) anti-GAPDH (p=0.0137). GAPDH is a loading control. All graphs are presented with Mean ± SEM]. Fig. S3. [Expression profile of CD63 in PD patients (P1-P4) and healthy controls (C1-C3). (A) Western blot of anti-CD63 and anti-GAPDH with equal sample volume (5µl) in PD and HC. Densitometric analysis of (B) anti-CD63 (p=0.0017) and (C) anti-GAPDH (p=0.047). GAPDH is a loading control. All graphs are presented with Mean ± SEM]. Fig. S4. [Expression profile of Flotillin-1 in PD patients (P1-P4) and healthy controls (C1-C3). (A) Western blot of anti- Flotillin-1 and anti-GAPDH with equal sample volume (5µl) in PD and HC. Densitometric analysis of (B) anti-Flotillin-1 (p=0.0213) and (C) anti-GAPDH (p=0.0448). GAPDH is a loading control. All graphs are presented with Mean ± SEM]. Fig. S5. [Expression profile of L1CAM in PD patients (P1-P4) and healthy controls (C1-C3). (A) Western blot of anti-L1CAM and anti-GAPDH with equal sample volume (5µl) in PD and HC. Densitometric analysis of (B) anti-L1CAM (p=0.0253) and (C) anti-GAPDH (p=0.0399). GAPDH is a loading control. All graphs are presented with Mean ± SEM]. Fig. S6. [Expression profile of Phospho-α-Synuclein in PD patients (P1-P4) and healthy controls (C1-C3). (A) Western blot of anti-phospho-α-Synuclein and anti-GAPDH with equal sample volume (5µl) in PD and HC. Densitometric analysis of (B) anti- phospho-α-Synuclein (p=0.0093) and (C) anti-GAPDH. GAPDH is a loading control. All graphs are pre [file 12916_2023_3031_MOESM1_ESM.docx]

**ADDITIONAL FILE 1 (Fig. S1-S8)**


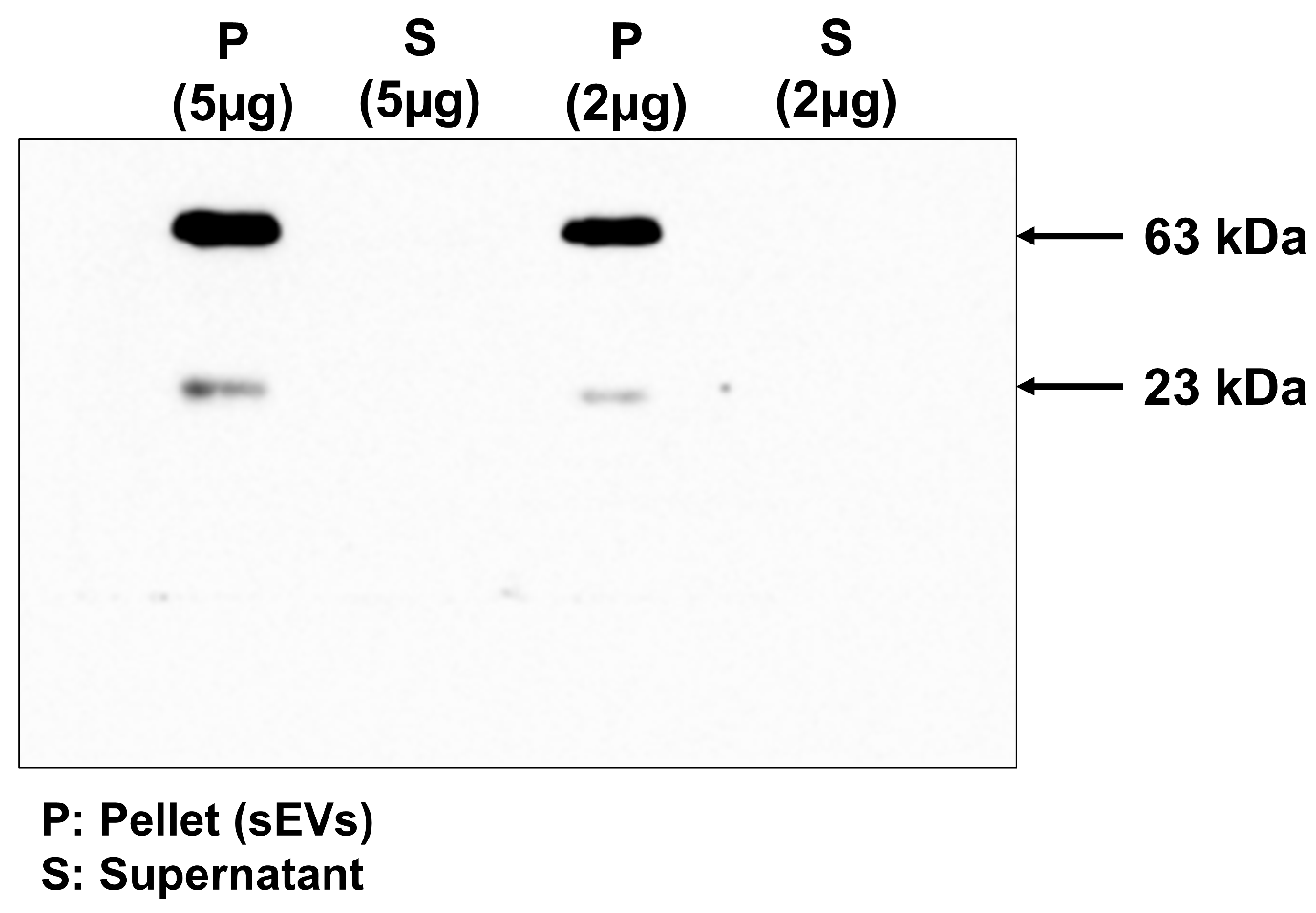


**Figure S1:** **Western blot of anti-CD63.** In this blot, the pellet obtained during the sEV isolation method and the supernatant, show the purity of sEVs. P= Pellet obtained by the PEG-based precipitation combined with ultrafiltration. S= Supernatant obtained during the isolation process.


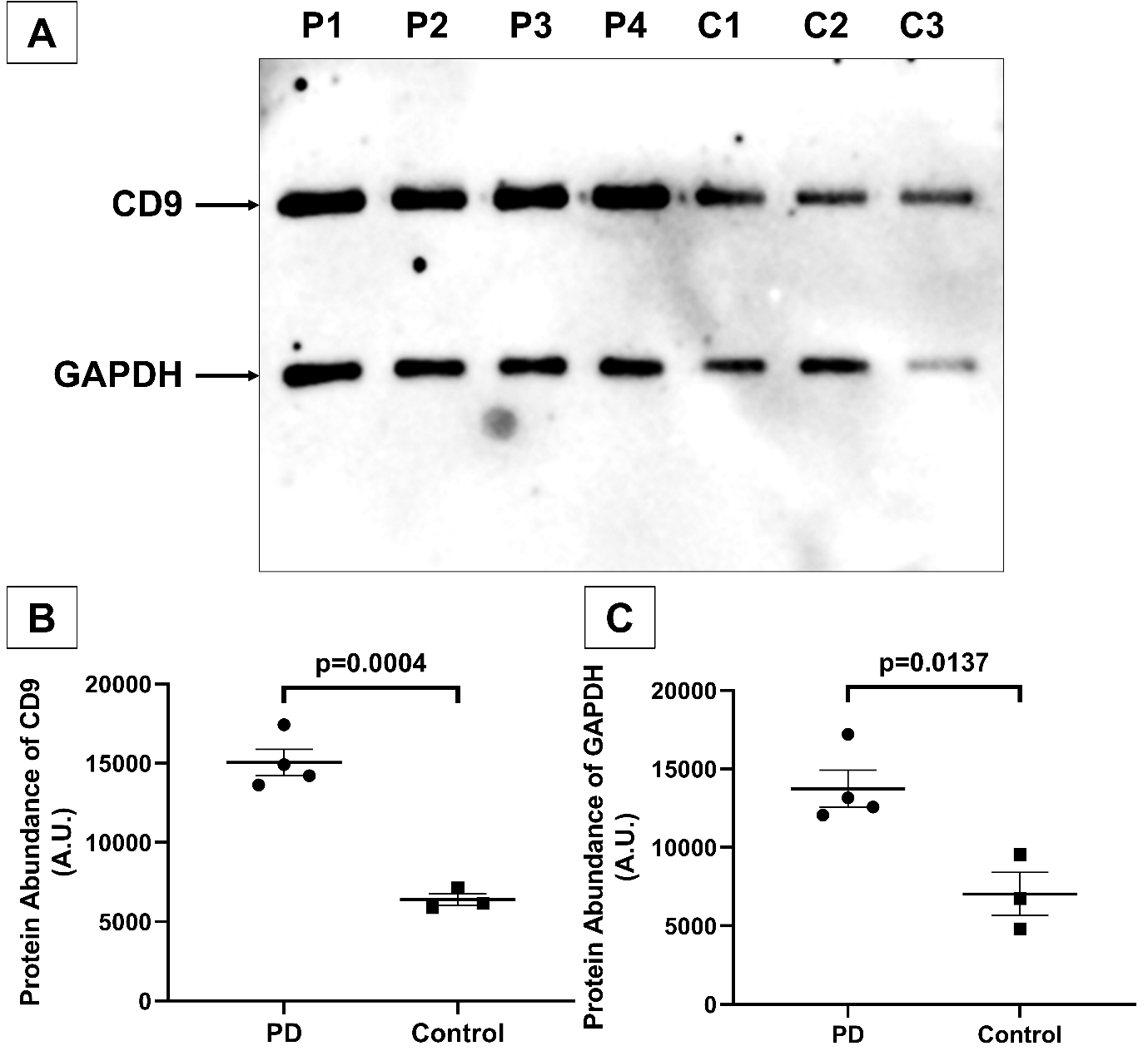


**Figure S2: Expression profile of CD9 in PD patients (P1-P4) and healthy controls (C1-C3). (A)** Western blot of anti-CD9 and anti-GAPDH with equal sample volume (5µl) in PD and HC. Densitometric analysis of **(B)** anti-CD9 (p=0.0004) and **(C)** anti-GAPDH (p=0.0137). GAPDH is a loading control. All graphs are presented with Mean ± SEM.


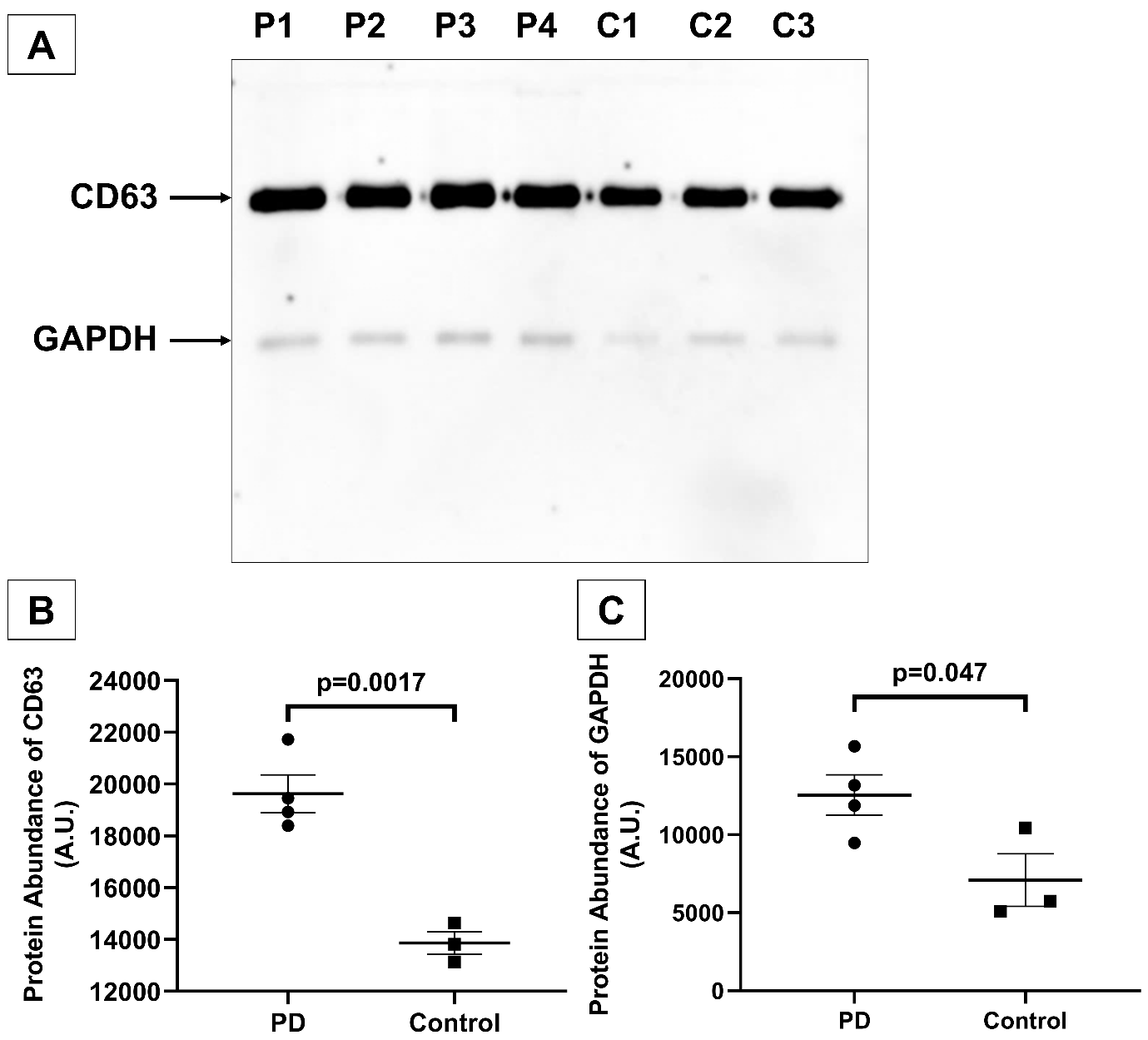


**Figure S3: Expression profile of CD63 in PD patients (P1-P4) and healthy controls (C1-C3). (A)** Western blot of anti-CD63 and anti-GAPDH with equal sample volume (5µl) in PD and HC. Densitometric analysis of **(B)** anti-CD63 (p=0.0017) and **(C)** anti-GAPDH (p=0.047). GAPDH is a loading control. All graphs are presented with Mean ± SEM.


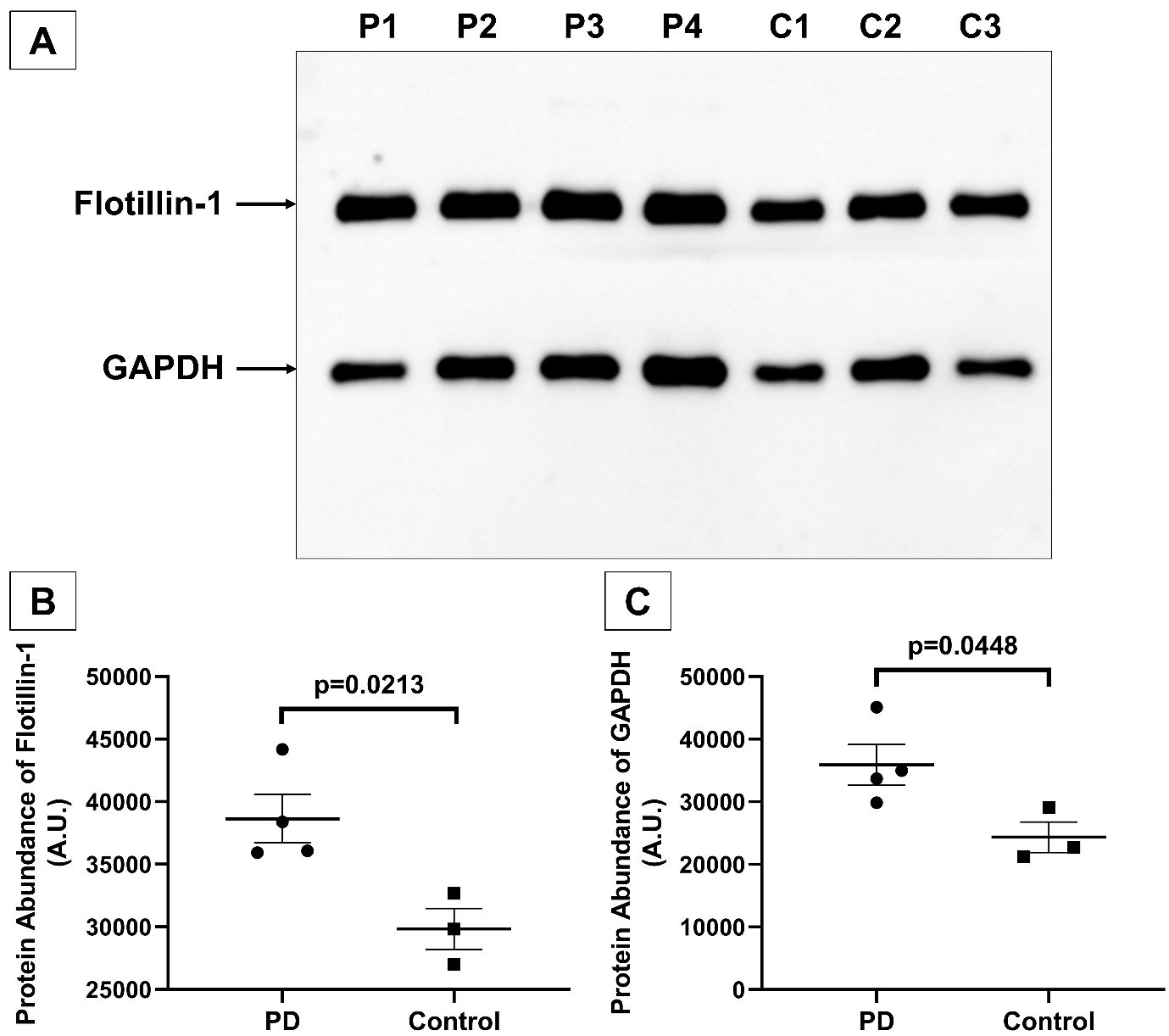


**Figure S4: Expression profile of Flotillin-1 in PD patients (P1-P4) and healthy controls (C1-C3). (A)** Western blot of anti- Flotillin-1 and anti-GAPDH with equal sample volume (5µl) in PD and HC. Densitometric analysis of **(B)** anti-Flotillin-1 (p=0.0213) and **(C)** anti-GAPDH (p=0.0448). GAPDH is a loading control. All graphs are presented with Mean ± SEM.


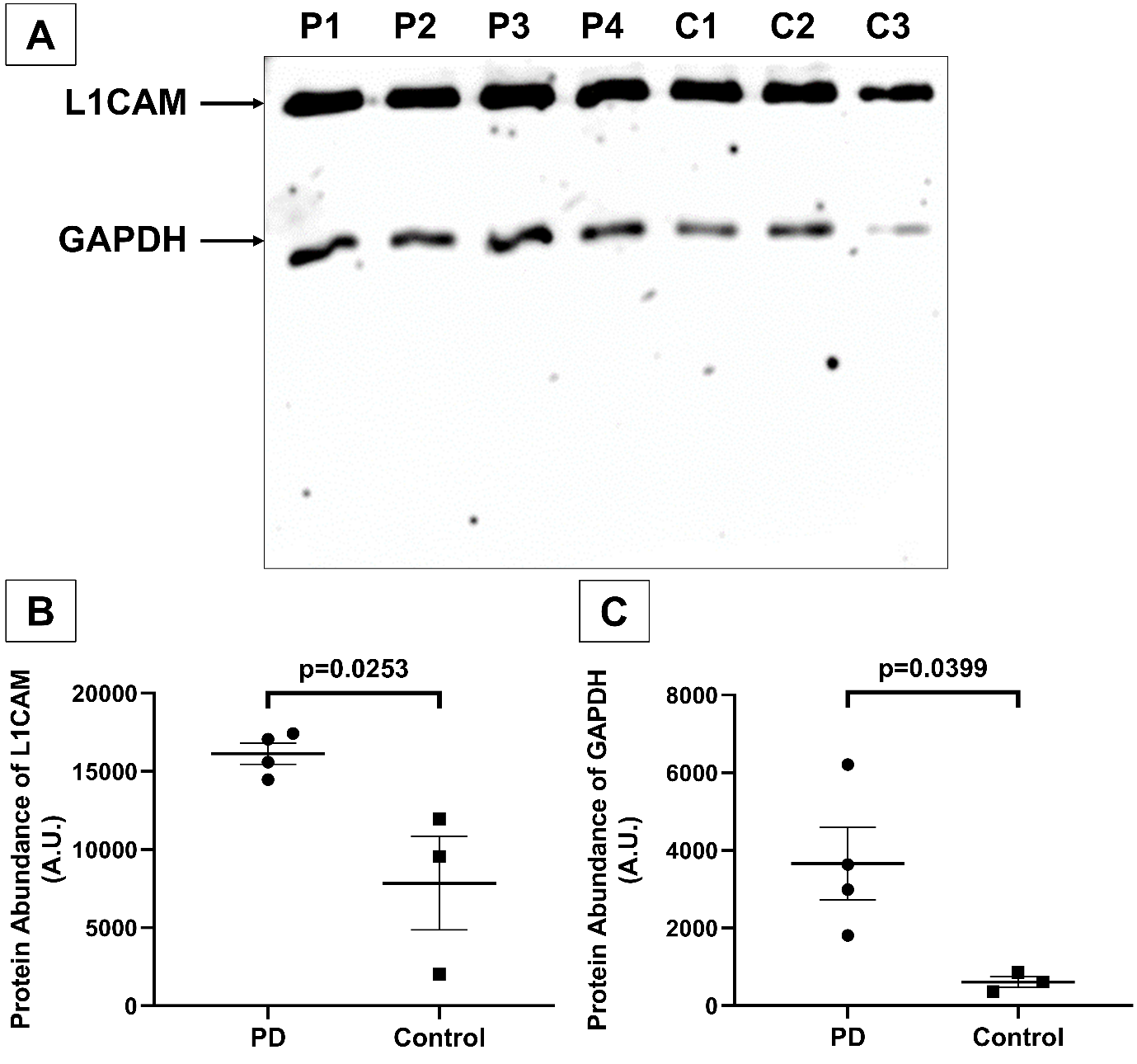


**Figure S5: Expression profile of L1CAM in PD patients (P1-P4) and healthy controls (C1-C3). (A)** Western blot of anti-L1CAM and anti-GAPDH with equal sample volume (5µl) in PD and HC. Densitometric analysis of **(B)** anti-L1CAM (p=0.0253) and **(C)** anti-GAPDH (p=0.0399). GAPDH is a loading control. All graphs are presented with Mean ± SEM.


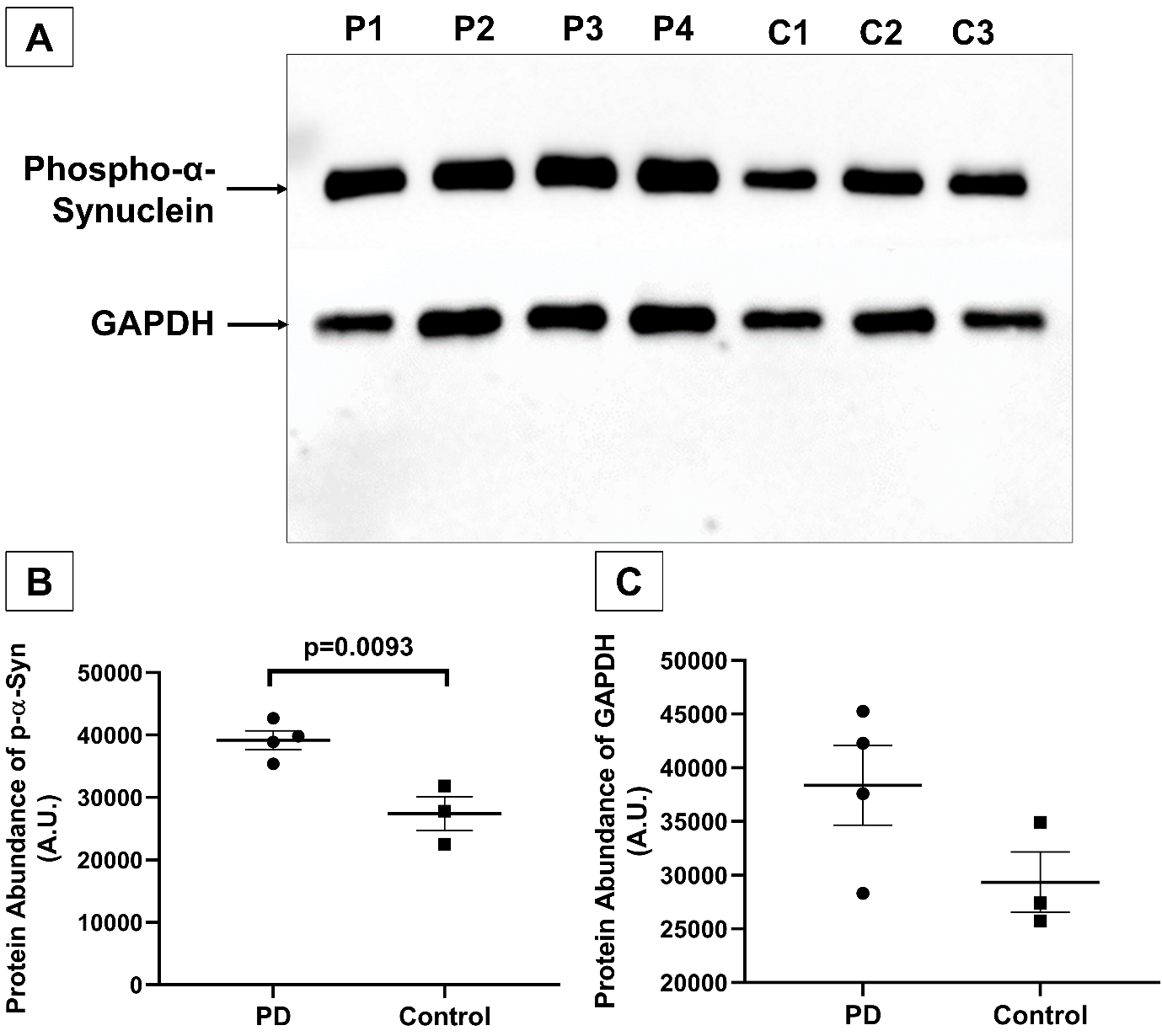


**Figure S6: Expression profile of Phospho-α-Synuclein in PD patients (P1-P4) and healthy controls (C1-C3). (A)** Western blot of anti-phospho-α-Synuclein and anti-GAPDH with equal sample volume (5µl) in PD and HC. Densitometric analysis of **(B)** anti- phospho-α-Synuclein (p=0.0093) and **(C)** anti-GAPDH. GAPDH is a loading control. All graphs are presented with Mean ± SEM.


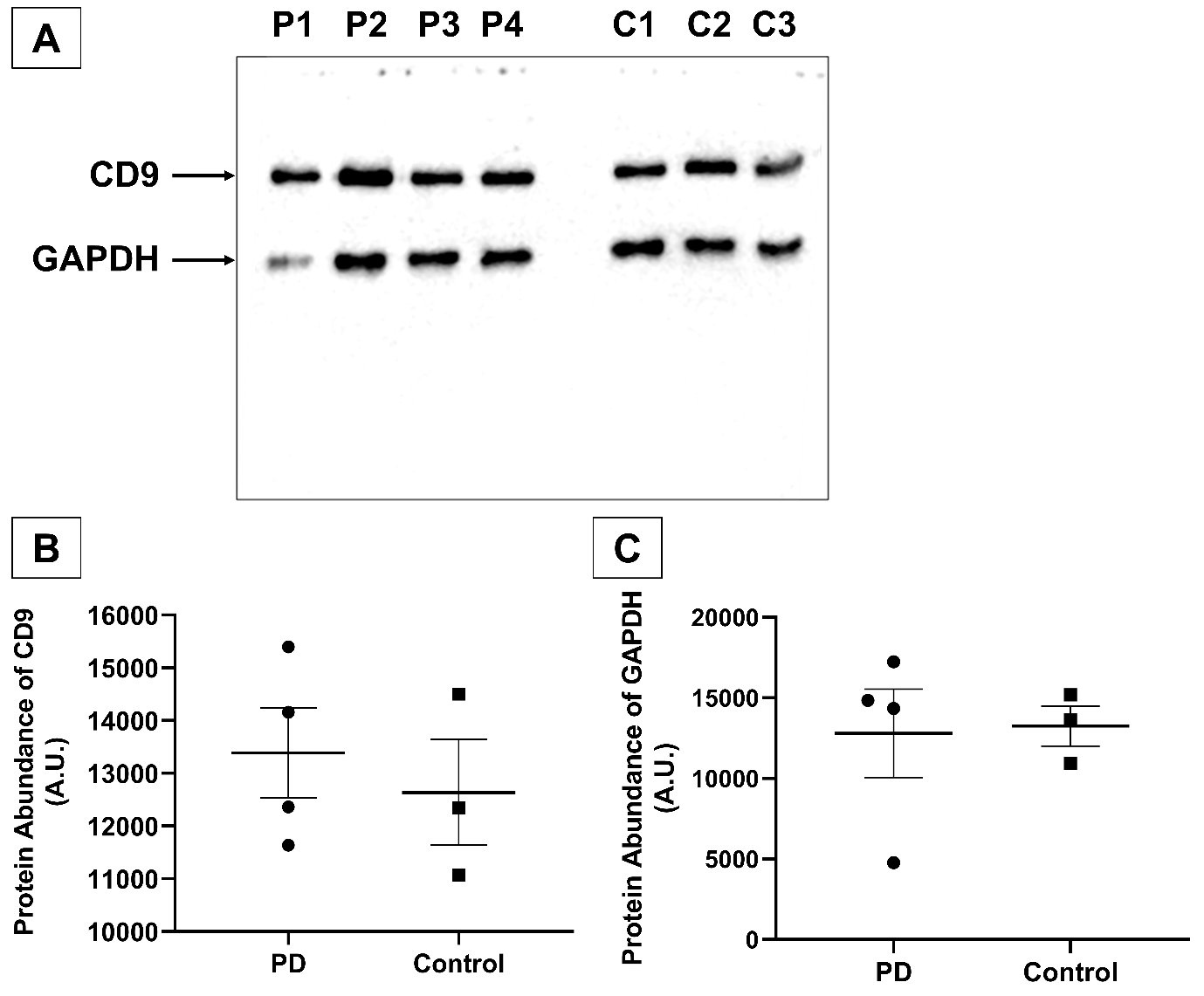


**Figure S7: Expression profile of CD9 with Concentration Normalization in PD patients (P1-P4) and healthy controls (C1-C3). (A)** Western blot of anti-CD9 and anti-GAPDH with equal protein sample loading (2.5µg) in PD and HC. Densitometric analysis of **(B)** anti-CD9 and **(C)** anti-GAPDH. GAPDH is a loading control. We did not observe significant differences in CD9 and GAPDH expressions in PD and HC. All graphs are presented with Mean ± SEM.

**
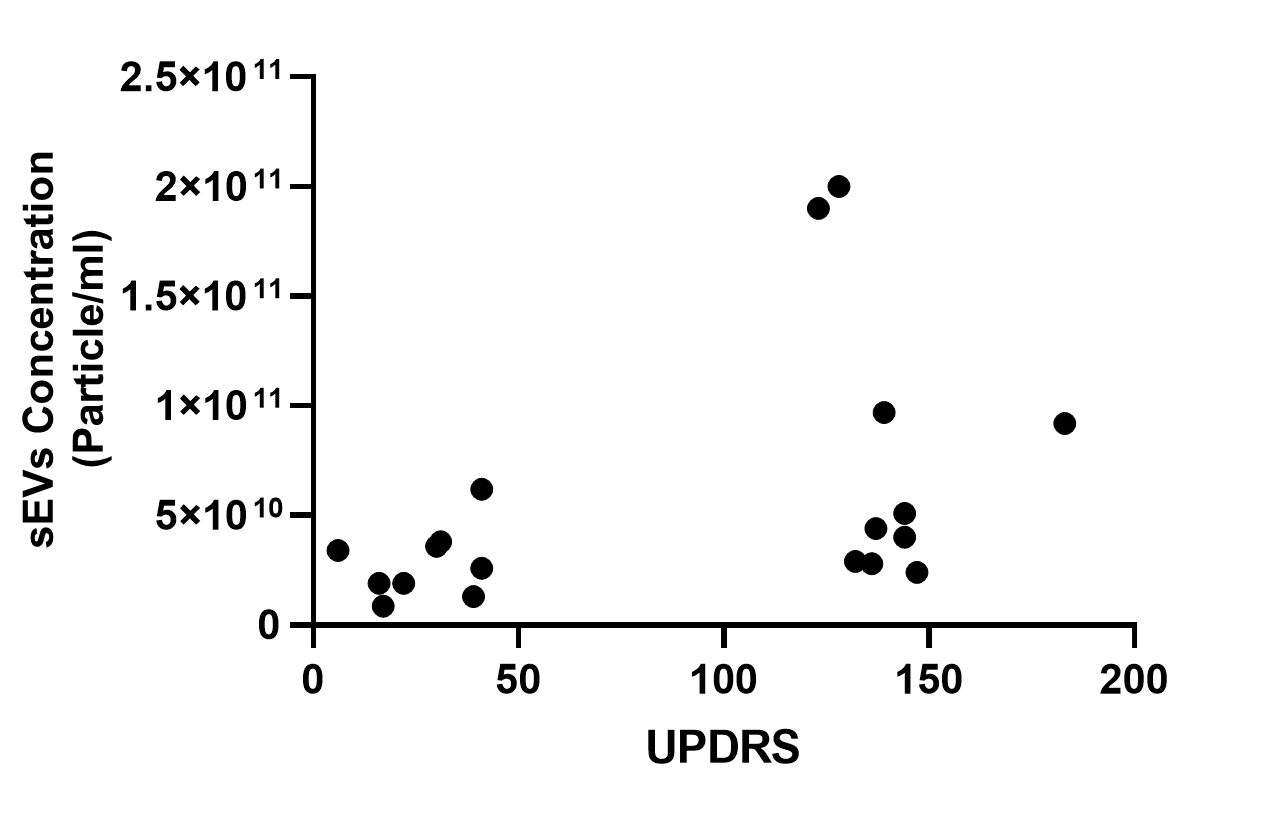
**

**Figure S8: Correlation coefficient analysis between sEV concentration and UPDRS score in controls and PD patients.**
